# Fully Implantable Wireless Cardiac Pacing and Sensing System Integrated with Hydrogel Electrodes

**DOI:** 10.1002/advs.202401982

**Published:** 2024-09-29

**Authors:** Zhiqiang Chang, Bingfang Wang, Qinjuan Ren, Jianfang Nie, Bihan Guo, Yuhan Lu, Xinxin Lu, Ya Zhang, Daizong Ji, Yingying Lv, Menahem Y. Rotenberg, Yin Fang

**Affiliations:** ^1^ Research Center for Translational Medicine Medical Innovation Center and State Key Laboratory of Cardiology Shanghai East Hospital The Institute for Biomedical Engineering & Nano Science Tongji University School of Medicine Shanghai 200120 China; ^2^ Research Centre of Nanoscience and Nanotechnology College of Science Shanghai University Shanghai 200444 China; ^3^ Department of Biomedical Engineering Technion – Israel Institute of Technology Haifa 32000 Israel

**Keywords:** cardiac pacing system, conductive hydrogel electrode, implantable, wireless and battery‐free

## Abstract

Cardiac pacemakers play a crucial role in arrhythmia treatment. Existing devices typically rely on rigid electrode components, leading to potential issues such as heart damage and detachment during prolonged cardiac motion due to the mechanical mismatch with cardiac tissue. Additionally, traditional pacemakers, with their batteries and percutaneous leads, introduce infection risks and limit freedom of movement. A wireless, battery‐free multifunctional bioelectronic device for cardiac pacing is developed. This device integrates highly conductive (160 S m^−1^), flexible (Young's modulus of 80 kPa is similar to that of mammalian heart tissue), and stretchable (270%) soft hydrogel electrodes, providing high signal‐to‐noise ratio (≈28 dB) electrocardiogram (ECG) recordings and effective pacing of the beating heart. The versatile device detects physiological and biochemical signals in the cardiac environment and allows for adjustable pacing in vivo studies. Remarkably, it maintained recording and pacing capabilities 31 days post‐implantation in rats. Additionally, the wireless bioelectronic device can be fully implanted in rabbits for pacing. By addressing a major shortcoming of conventional pacemakers, this device paves the way for implantable flexible bioelectronics, which offers promising opportunities for advanced cardiac therapies.

## Graphical abstract

A wireless, battery‐free multifunctional bioelectronic device for cardiac pacing is developed, integrating highly conductive, flexible, and stretchable hydrogel electrodes. The multifaceted device seamlessly integrates the detection of physiological signals with the analysis of biochemical markers indicative of heart disease, thereby facilitating adaptable cardiac disease management strategies during in vivo investigations.

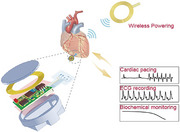

## 1 Introduction

Implantable pacemakers effectively treat bradyarrhythmias, but traditional devices often necessitate extracorporeal wiring post‐implantation, which is prone to detachment, breakage, and poses systemic infection risks.^[^ ^1^ ^]^ Moreover, the dependency on battery power sources introduces drawbacks such as large size, rigidity, and a limited lifespan, necessitating repeated, high‐risk surgical replacements every 5–10 years, which incur significant financial and health burdens.^[^ ^2^ ^]^ Recent advancements in battery‐free cardiac pacemakers, utilizing external wireless power^[^ ^3^ ^]^ and friction‐based energy harvesting,^[^ ^4^ ^]^ have shown promise in enhancing longevity. Furthermore, cardiac pacemaker implantation can lead to complications, including heart failure. In situ physiological and biochemical monitoring has received heightened interest due to its importance in providing immediate and pertinent cardiac health feedback during pacemaker implantation. Nonetheless, this monitoring necessitates reliable and continuous electrical power. Consequently, demand for wireless, battery‐free pacemakers that consolidate physiological and biochemical monitoring within a unified system is growing.^[^ ^5^ ^]^

Optimal implant electrodes should mimic the mechanical softness and deformability of cardiac tissue. Yet, conventional rigid electrodes are associated with complications like infections and inflammation due to the mechanical mismatch with the soft, curvilinear, and dynamic cardiac environment.^[^ ^6^ ^]^ Conductive hydrogels have emerged as superior alternatives to metal electrodes for bioelectronic interfaces, finding applications in cardiac electrophysiology,^[^ ^7^ ^]^ soft tissue adhesives^[^ ^8^ ^]^, and neuromodulation,^[^ ^9^ ^]^ owing to their tissue‐like water content, electrical conductivity, and flexibility. However, the prevalent method for integrating conductive polymers into hydrogel matrices often undermines hydrogel's mechanical integrity,^[^ ^10^ ^]^ limiting its bioelectronic interface performance. Currently, research on hydrogel electrodes for sensing and stimulation typically involves implanting the electrodes into the body, routing leads externally, and connecting them to external devices for working.^[^ ^1^, ^3^, ^7^, ^11^ ^]^ The integration of hydrogel electrodes with implantable cardiac sensing and pacing electronic systems has not been achieved, posing challenges such as instability and the potential for infection and inflammatory responses. Designing durable and flexible conductive hydrogel electrodes that balance electrical and mechanical properties integrated with implantable cardiac pacing and monitoring systems is imperative.

**Figure 1:**
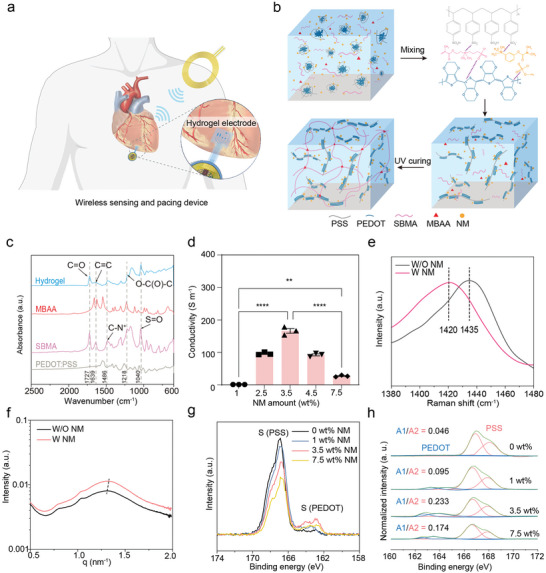
Hydrogel design for enhanced electrical conductivity and stretchability. a) Schematic representation illustrating a multifunctional wireless bioelectronic device with integrated hydrogel electrodes. b) The schematic diagram illustrates the structural composition of the internal network within the hydrogel and the interactions between molecules. c) FTIR spectra for individual components and the complete hydrogel, highlighting characteristic peaks of SBMA, MBAA, and PEDOT: PSS. d) Conductivity of the hydrogels doped with varying NM concentrations (*n* = 3). *p < 0.05, **p < 0.01, ***p < 0.001, and ****p < 0.0001. e) Raman shift of hydrogels with NM, indicating a transition in PEDOT: PSS from benzoid to quinoid structures. f) SAXS 1D curves for hydrogels, comparing samples with and without NM. g) XPS profiles of the hydrogel surface, revealing a reduction in PSS content as NM concentration increases. h) The peak‐fitting of the XPS profiles and the variation of PEDOT to PSS ratio as the NM content increases in the hydrogel.

In this study, we developed a miniaturized multifunctional wireless sensing and pacing device (WSPD), which integrates highly conductive (160 S m^−1^), stretchable (270%), and flexible (80 kPa) hydrogel electrodes (**Figure** **1**a). This externally programmable device delivers cardiac pacing in response to detected abnormal sinus rhythms and incorporates drivers for biochemical sensors and modules for biochemical signal detection. This integration enables in situ biochemical signal monitoring alongside flexible transistor sensors, providing a comprehensive view of cardiac health. Utilizing coil resonant coupling for power transfer, the WSPD overcomes the battery life limitations. The device demonstrates the dual capability of ECG and biochemical signal detection and applies its therapeutic potential for arrhythmia treatment, which is validated through ex and in vivo studies. Notably, the WSPD maintains operational effectiveness over 31 days of implantation, demonstrating the ability to perform intra‐body cardiac pacing in small to medium‐sized animals. The integration of conductive hydrogel electrodes with cardiac sensing and stimulation electronic systems represents a groundbreaking technological advancement in the treatment of heart diseases.

## 2 Results and Discussion

Our hydrogel system utilized poly(3,4‐ethylenedioxythiophene) polystyrene sulfonate (PEDOT:PSS) as the conductive medium. However, PEDOT: PSS alone is suboptimal due to its inherent structural limitations, resulting in suboptimal conductivity and tensile strength.^[^ ^12^ ^]^ To address these issues, we included a UV‐reactive zwitterionic monomer, [2‐(methacryloyloxy) ethyl] dimethyl‐(3‐sulfopropyl) ammonium hydroxide (SBMA), to strengthen the mechanical network (Figure 1b). Additionally, we incorporated the ionic additive neostigmine methanesulphate (NM) to boost the hydrogel's electrical properties.

During the initial setting of the hydrogel, NM disrupts the interaction between PEDOT and PSS via electrostatic shielding.^[^ ^13^ ^]^ This disruption allows PEDOT chains to stretch and form tightly packed π‐π stacks, enhancing the material's connectivity and crystalline structure. We utilized Fourier transform infrared (FTIR) spectroscopy to confirm the formation of the covalent crosslinking network within the hydrogels(Figure 1c). The FTIR spectra revealed characteristic peaks of the SBMA component at specific wavenumbers: 1040 cm^−1^ for S═O asymmetric stretching, 1218 cm^−1^ for ─O─C(O)─C stretching, 1486 cm^−1^ for C─N stretching, 1639 cm^−1^ for C═C stretching, and 1727 cm^−1^ for C═O stretching.^[^ ^14^ ^]^ These peaks indicated the successful incorporation of SBMA into the hydrogel network.

The hydrogel's conductivity was affected by the concentration of NM. As shown in Figure 1d, the conductivity initially increased with additional NM, peaking at 160 S m^−1^ at a NM concentration of 3.5 wt.%, markedly higher than that of biological tissues (0.3–0.7 S m^−1^) and other conducting hydrogels, such as PEDOT: PSS/PVA hydrogel (9 S m^−1^)^[^ ^15^ ^]^ (Table S1, Supporting Information). Beyond this concentration, conductivity began to decline. We hypothesize that this decrease is due to the interaction between NM anions and PEDOT, which may interfere with the orderly stacking of PEDOT chains, thus diminishing the hydrogel's conductivity.^[^ ^16^ ^]^

We analyzed the molecular state of PEDOT‐rich domains using Raman spectroscopy. The addition of NM led to a redshift in the C_α_ = C_β_ vibrational peak (≈1435 cm^−1^) in the Raman spectrum (Figure 1e), indicating the transformation of PEDOT molecules from a benzoid to a more conductive quinoid structure. The Raman peak fitting results showed an increase in the quinoid‐to‐benzoid structure ratio after NM was added (Figures S1 and S2, Supporting Information),^[^ ^17^ ^]^ suggesting a more elongated PEDOT chain conducive to enhanced electrical conductivity. Small‐angle X‐ray scattering (SAXS) results showed that the average distance between PEDOT‐rich domains decreased with more NM, implying tighter stacking and thus enhanced conductivity (Figure 1f; Figure S3, Supporting Information).^[^ ^18^ ^]^ X‐ray photoelectron spectroscopy (XPS) analysis further confirmed that as the NM concentration increased from 0 wt% to 3.5 wt% (Figure 1g,h), the PEDOT/PSS ratio also increased, peaking at a value of 0.233 at a NM concentration of 3.5 wt%. This result indicated that a large portion of insulating PSS was removed with the increase of NM.^[^ ^19^ ^]^ Beyond this concentration, the PEDOT/PSS ratio began to decline, consistent with the observed trend in conductivity.

In terms of biocompatibility, our hydrogels perform exceptionally well, which is an essential attribute for their application in bioelectronics. In vitro cytotoxicity assessments showed that both human embryonic kidney 293 (HEK293) cells and cardiomyocytes cultured in a medium containing hydrogel extracts exhibited survival rates comparable to the control group, suggesting that the hydrogel has no significant toxicity^[^ ^20^ ^]^ (Figures S4 and S5, Supporting Information). Additionally, to explore in vivo biocompatibility, we implanted the hydrogel and platinum (Pt) electrodes into the subcutaneous tissue of the backs of rats for a week, respectively. After pathological analysis of hematoxylin and eosin (H&E) staining according to “ISO 10993–6:2016” (Table S2, Supporting Information), the conductive hydrogel produced an inflammatory effect close to that of the control group (Pt), showing a mild inflammatory response, indicating its significant degree of biocompatibility (Figure S6, Supporting Information).^[^ ^21^ ^]^

**Figure 2:**
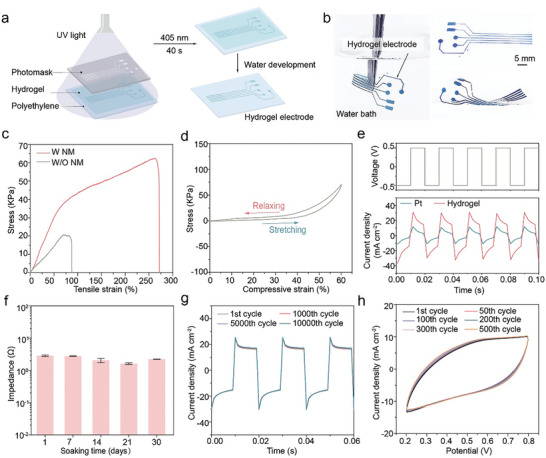
Lithographically patterned hydrogel and its mechanical and electrical properties. a) Schematic of the hydrogel electrode patterning process using photolithography, where UV light through a photomask enables selective patterning, followed by development in deionized water. b) Optical images showcasing the microstructured hydrogel electrodes, with the left image highlighting the electrode's flexibility in water. c) Tensile properties of hydrogel electrodes with and without NM, hydrogels with NM can be elongated up to 270%. d) Mechanical compression properties of the hydrogels with NM. e) Current density versus time plots comparing hydrogel electrode and Pt electrode when subjected to a voltage of ±0.5 V with a pulse width of 10 ms, across an area of 0.25 cm^2^. f) Impedance measurements of hydrogel electrodes at 1 kHz after a 30 days of PBS soak (*n* = 3). g) Current density versus time graph during 10000 voltage pulses with a 10 ms pulse width and a voltage of ±0.5 V applied over a 0.3 cm^2^ area, demonstrating the cyclic stimulation durability of the hydrogel electrode. h) CV curves of the hydrogel electrode in PBS.

Our flexible hydrogel electrodes can be precisely patterned using direct photolithography (**Figure** **2**a). The mild lithography process preserved the hydrogels' softness and flexibility (Figure 2b). These hydrogels showed impressive mechanical properties, stretching up to 270% under tensile stress while maintaining a low Young's modulus of 80 kPa (Figure 2c). The Young's modulus of the hydrogel electrodes is close to that of mammalian hearts,^[^ ^22^ ^]^ which is significantly lower than that of traditional metal electrodes (63 GPa for Ag, 102 GPa for Cu, and 57 GPa for Al),^[^ ^23^ ^]^ demonstrating the softness of hydrogel electrodes. This hydrogel shows higher tensile properties and lower Young's modulus than other composites, such as PEDOT: PSS/DMSO hydrogel (35%)^[^ ^10b^ ^]^ (Figure S7; Table S1, Supporting Information). It exhibits reversible elasticity, even after 60% compression (Figure 2d), allowing it to conform to the heart's dynamic mechanical movements. Remarkably, the conductivity of our hydrogels is minimally affected by strain, capable of powering a light bulb even when stretched to about double its original length (Figure S8, Supporting Information). The hydrogel exhibits exceptional impedance stability even under 100% tensile deformation (Figure S9, Supporting Information). Moreover, after enduring 5000 cycles of 35% tensile stretching, its impedance remains virtually unchanged, demonstrating its outstanding tensile stability (Figure S10, Supporting Information).

The comparison between the hydrogel and the Pt electrodes on curved surfaces demonstrated that hydrogel electrodes adapt better to highly curved surfaces with their conformability (Figure S11, Supporting Information). Pt electrodes, with a maximum allowable strain of only 3.4%,^[^ ^24^ ^]^ are unsuitable for applications involving continuous heartbeats. By simulating the systole and diastole processes of heartbeats through balloon inflation and deflation, we observed that the hydrogel adhered well and exhibited excellent deformation compliance (Figure S12; Movie S1, Supporting Information). This optimal adhesive properly ensures intimate contact between the electrode and heart interfaces for stable and accurate signal transmission. We conducted a 180° peel test to measure the interfacial toughness and evaluate the adhesive performance of the hydrogel on various substrates (including glass slides, PI, and PET).^[^ ^25^ ^]^ Additionally, we applied the hydrogel to an ex vivo heart to observe its adhesion (Figure S13, Supporting Information). The results indicated that the conductive hydrogel exhibited excellent adhesive properties on these substrates, which was crucial for maintaining a stable interface with heart tissue during cardiac pacing and monitoring.

We also investigated the hydrogels' electrochemical characteristics. They show a superior charge injection capacity (CIC) of 18.6 mC cm^−2^ at voltage pulses of ±0.5 V (Figure 2e), nearly tripling the CIC of bare Pt electrodes. Compared to bare Pt electrodes, hydrogel electrodes exhibit lower electrochemical impedance spectroscopy (EIS) and higher charge storage capacity (CSC), as demonstrated by various hydrogel compositions (Figure S14, Supporting Information). To assess the electrical stability of the hydrogel‐heart interface, we attached the hydrogel electrodes to rat hearts and pacing with 3.3 V, and 5 Hz pulses while recording the ECG signal amplitude over a prolonged period. The experimental results showed that after 15 000 pulses (Figure S15, Supporting Information), the ECG signal amplitude remained essentially unchanged, indicating the excellent electrical performance of the hydrogel electrode at the heart interface. The hydrogel electrode demonstrated favorable electrical stability when compared to the Pt electrode.

For in vivo cardiac pacing, the electrical stability of the hydrogel interface is paramount. Our hydrogels maintained a stable conductivity of ≈70 S m^−1^ after 8 weeks in water (Figure S16, Supporting Information) and showed no significant change in EIS and CSC after a month in phosphate‐buffered saline (PBS) (Figure 2f; Figure S17, Supporting Information). Moreover, the hydrogels exhibited excellent cyclic stability, withstanding 10000 cycles for charging and discharging (Figure 2g). The cyclic voltammetry (CV) curves exhibited consistent behavior over numerous cycles, with minimal variations in CSC (Figure 2h; Figure S18, Supporting Information), affirming the hydrogels' suitability as implantable electrodes, especially in a solution mimicking bodily fluids.

**Figure 3:**
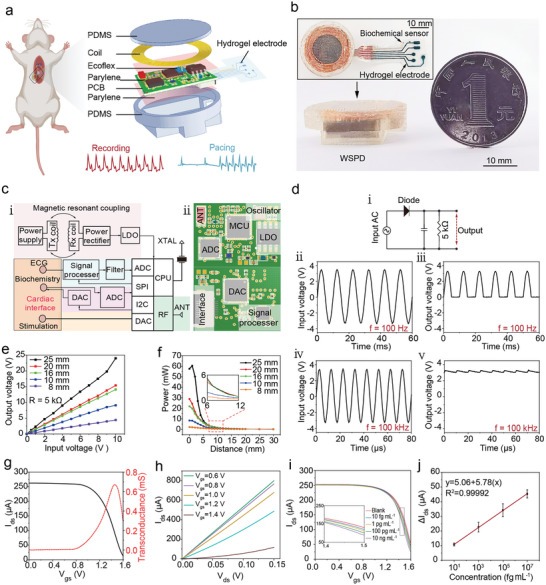
Design and validation of the WPSD. a) Illustration depicting the structural composition and encapsulation of the WSPD. b) Photographs of the WSPD, including wireless power supply devices and flexible electrodes. The flexible electrodes comprise hydrogel electrodes and biochemical sensors. c) i: Electrical schematic diagram of the wireless power supply device. ii: Schematic diagram of the PCB structure featuring components such as MCU, ADC, DAC, LDO, and signal process chip. d) Schematic diagram demonstrating the operation of the infinite rectifier circuit at varying frequencies. e) Changes in output voltage for different sizes of Rx coils when subjected to input voltage from the Tx coils, with a 5 kΩ load, and the distance between the transmitting and receiving coils is 1 mm. f) Variation of output power with transmission distance for different sizes of Rx coils, with an input voltage of 10 V and a load of 5 kΩ. g) OECT transfer characteristic curve and transconductance curve with the solution as the dielectric layer. h) OECT output characteristic curve with the solution as the dielectric layer (*n* = 3). i) Transfer characteristic curve for NT‐proBNP concentrations in the range of 10 fg mL^−1^–10 ng mL^−1^. j) Calibration curve obtained from i (*n* = 3).

We developed an implantable WSPD integrated with the conductive and stretchable hydrogel electrodes. This multifunctional device is designed to deliver in situ cardiac pacing, capture real‐time ECG signals, and monitor biochemical signals (Table S3, Supporting Information). **Figure** **3**a illustrates the intricate design of the device, which incorporates a 30 µm‐thick parylene coating on the printed circuit board (PCB) for moisture and humidity resistance. The device was further encapsulated in 1 mm‐thick biocompatible polydimethylsiloxane (PDMS), thus solving the problem of the modulus mismatch between the hydrogel electrodes and the PCB, and ensuring reliable and stable performance after implantation. We designed gold electrodes on a flexible substrate to bridge the connection of the PCB. A portion of the gold electrodes was connected to the conductive hydrogel electrodes, encapsulated in Ecoflex for protection. The other side was connected to the biointerface of the PCB, ensuring electrical continuity between the electronic system and the hydrogel electrodes (Figure 3b).^[^ ^26^ ^]^ The WSPD is composed of four essential modules (detailed in Figure 3c): 1. a wireless power supply module that operates through resonant inductive coupling between coils, ensuring the device remains powered without direct electrical connections; 2. a cardiac pacing module that allows for precise adjustments of the pacing frequency and pulse width, catering to the specific needs of the heart pacing; 3. a dual‐function module that not only detects real‐time ECG signals but also performs in situ electrochemical signal detection, providing comprehensive cardiac monitoring; 4. an antenna module that enables seamless wireless communication with external devices, such as mobile phones, for data transmission and device control. Compared to other cardiac pacemakers, the complex configuration of WSPD enables in situ physiological and biochemical monitoring of the heart while simultaneously providing cardiac pacing therapy. This paves the way for advanced cardiac treatment strategies.

Unlike traditional battery‐powered systems, this device uses coil coupling for power supply. Therefore, the wireless power transfer module within the device is crucial. The block diagram in Figure 3c outlines the electrical system of the WSPD. The process begins with the receiver (Rx) coil, which picks up a 220 kHz radio frequency (RF) signal from an external transmitting (Tx) coil. Compared to other power sources like ultrasound and triboelectric generators, RF coupling not only meets the power consumption needs of our device but also significantly reduces its size (diameter is 30 mm) (Table S3, Supporting Information). Additionally, 220 kHz RF signals offer higher safety compared to higher frequency RF signals, minimizing the impact on the animals.^[^ ^27^ ^]^ This RF signal induces an alternating current (AC) in the Rx coil. The AC is then converted into a direct current (DC) through a half‐wave rectifier circuit. This pulsating DC signal is then regulated to a steady 3.3 V DC output using a low dropout (LDO) linear regulator providing a stable power DC supply for the integrated circuits of the WSPD.

For the ECG signal acquisition, the WSPD integrates the analog‐to‐digital conversion (ADC) channel of a low‐power microcontroller unit (MCU). The ADC captures the analog ECG signal and converts it into a digital format for processing. In parallel, the MCU interfaces with a signal processing chip using the serial peripheral interface (SPI) protocol. This allows the ECG signal to be amplified and filtered, preparing it for transmission. The processed ECG signal is then wirelessly transmitted to an external mobile device using an RF antenna at 2.4 GHz. (Figures S19–S21, Supporting Information). The external device serves multiple purposes, including data storage, display, and analysis of the ECG signal. Moreover, it can send control signals back to the WSPD, adjusting cardiac pacing parameters such as the pacing frequency and pulse width. Figure 3d presumably illustrates the half‐wave rectifier circuit. At a lower frequency (100 Hz), the diode in the circuit allows only the positive portion of the AC signal to pass, creating a pulsating positive portion of the AC on the load side. However, at higher frequencies (above 100 kHz), the output closely approximates a continuous DC signal. Following this rectification, the voltage is regulated to 3.3 V DC by the LDO, ensuring a consistent power supply for the WSPD (Figure S22, Supporting Information).

To meet the requirements for implantation, we studied the effects of coil size and transmission distance on output power. We observed that the output power of the Rx coil positively correlates with its size and inversely with the distance to the Tx coil (Figure 3e,f). When the 25 mm diameter Rx coil was placed 10 mm away from the transmitting coil, it produced a power output of 2 mW. The MCU inside the WSPD controls the signal processing chip via a communication protocol for ECG signal collection. The time‐division multiplexing functionality of the protocol allows a single low‐power MCU to facilitate a modular design with different functionalities. In this context, we incorporated a biochemical detection module to further assess the cardiac health status. This module, controlled by the MCU, utilizes the Inter‐Integrated Circuit (I2C) communication protocol to command an external digital‐to‐analog converter (DAC) for voltage generation driving biochemical sensors. Simultaneously, an external ADC was used to collect biochemical signals, transmit them to the MCU, and finally send the collected biochemical signals to external devices via an RF antenna.

Subsequently, we incorporated a flexible organic electrochemical transistor (OECT) into the WSPD to detect N‐terminal pro‐B‐type natriuretic peptide (NT‐proBNP), a heart failure biomarker, and to modulate the pacing frequency in response to aberrant NT‐proBNP levels. We evaluated the OECT's transmission and output properties (Figure 3g,h). The repeatability of OECT was initially verified (Figure S23, Supporting Information). The response curves of the OECT to NT‐proBNP concentrations were then delineated, with a standard curve of current response relative to concentration constructed via statistical analysis (Figure 3i,j). The transistor exhibited a sensitive biological response to NT‐proBNP, with detection ranging from 10 fg mL^−1^ to 10 ng mL^−1^ (R^2^ = 0.99992).

**Figure 4:**
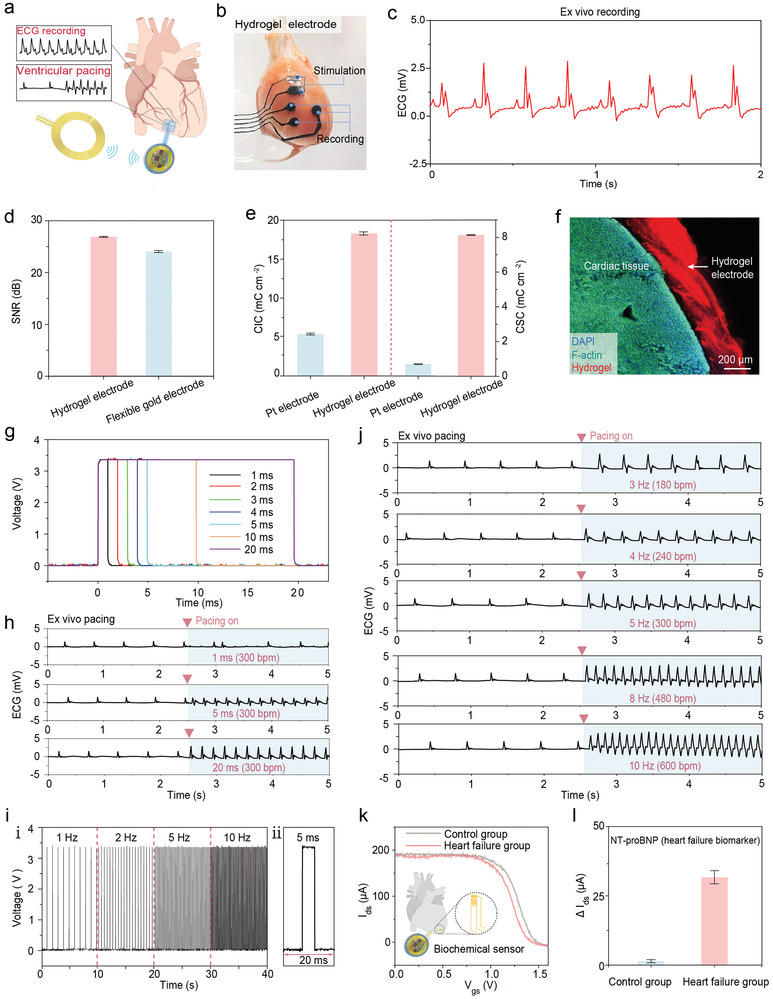
In vitro cardiac sensing and pacing. a) The schematic diagram illustrates the application of the WSPD in pacing and ECG recording on the ex vivo heart. b) The image depicts hydrogel electrodes adhered to the heart for ECG signal detection and cardiac pacing (*n* = 3). c) ECG signal detected by the integrated system in the ex vivo rat's heart (*n* = 3). d) SNR of ECG signals using hydrogel electrodes and flexible gold electrodes (*n* = 3). e) The statistical values of CIC and CSC between hydrogel electrodes and Pt electrodes (*n* = 3). f) Confocal image of hydrogels adhered onto the heart's surface, where the red‐stained portion represents the hydrogel electrode component. g) The device's ability to adjust the pulse width for cardiac pacing. h) Changes in the sinus rhythm of the ex vivo rat heart under stimulation with different pulse widths (*n* = 3). i) i: The device's capability to generate pulsed stimulation at various frequencies. ii: Various frequencies are tested with an amplitude of 3.3 V and a pulse width of 5 ms. j) The effect of different pacing frequencies on sinus rhythm in the ex vivo rat's heart (*n* = 3). k) The response curve detected by the OECT in the presence and absence of NT‐proBNP. The heart containing or lacking NT‐proBNP is used to simulate abnormal concentrations of the biomarker in heart failure (*n* = 3). l) Comparison of the current changes of the OECT with or without NT‐proBNP (*n* = 3).

The hydrogel electrodes‐integrated WSPD was validated for cardiac pacing, and ECG signal monitoring using ex vivo hearts. (**Figure** **4**a). The flexible hydrogel electrodes conformably adhere to the irregular shape of the heart surface (Figure 4b). The hydrogel electrodes of WSPD capture ECG signals (230 bpm) of ex vivo rat hearts (Figure 4c). These hydrogel electrodes exhibited a higher signal‐to‐noise ratio (SNR) during ECG recording compared to metal electrodes (Figure 4d; Figure S24, Supporting Information). This demonstrated that the hydrogel electrodes have superior resistance to interference, particularly in dynamic environments with continuous cardiac activity, thereby providing more stable and accurate ECG signals. Excellent charge storage and injection capabilities of hydrogel electrodes (Figure 4e) contribute to the efficient release of electrical pulses. The enhanced CIC enables more effective signal transmission between the electrode and heart tissue. Additionally, the stable and secure interface between the hydrogel electrodes and the heart is a crucial factor in achieving high SNR records of ECG signals. We examined this interface using scanning electron microscopy (SEM) and confocal imaging (Figure 4f; Figures S25 and S26, Supporting Information), which demonstrated the conformality of hydrogel electrodes to the heart's surface. This adaptability not only reduces the risk of cardiac damage but also ensures the stability of cardiac detection and pacing operations amidst continuous heartbeats (Movie S2, Supporting Information).

The integration of hydrogel electrodes into the WSPD enables effective cardiac pacing. Hydrogel electrodes act as intermediaries to regulate myocardial contractions by modifying the pulse width of electrical stimulation pulses (Figure 4g). The impact of different pulse widths on ex vivo rat heart cardiac pacing was illustrated in Figure 4h and Figure S27 (Supporting Information). No significant changes in heart rhythm were observed when the pulse width was less than 1 ms. However, when the pulse width falls below 5 ms affects the heart rate, though the amplitude remains suboptimal compared to the baseline. Effective cardiac pacing was only achieved when the pulse width surpassed 5 ms (Figure S28, Supporting Information). Additionally, the heart rate was able to be controlled by adjusting the pulse frequency of the electrical stimulation pulses (Figure 4i,j). For example, by stimulating an ex vivo rat's heart with arrhythmia using a pulse stimulation frequency of 10 Hz and an amplitude of 3.3 V, we observed an immediate widening and amplification of the QRS waveform in the ECG, resulting in a heart rate of 600 beats per minute (bpm). Therefore, our hydrogel electrodes can monitor and track the rat's cardiac rhythm and health while effectively providing pacing. The pacing effect can be adjusted by transmitting commands to the wireless pacing module through external control signal devices. To further validate the WSPD's capability in detecting biochemical signals, we established a heart failure model characterized by biomarker NT‐proBNP abnormalities. This built on our previous validation of the bioelectronic system's biochemical signal detection (Figure S29, Supporting Information). Antigen concentration testing under wireless‐powered mode revealed a significant decrease (≈30 µA) in source‐drain current as the NT‐proBNP concentration increased by 0.1 ng mL^−1^ (Figure 4k,l), indicating that the WSPD can power the flexible transistor to effectively detect heart failure biomarker.

**Figure 5:**
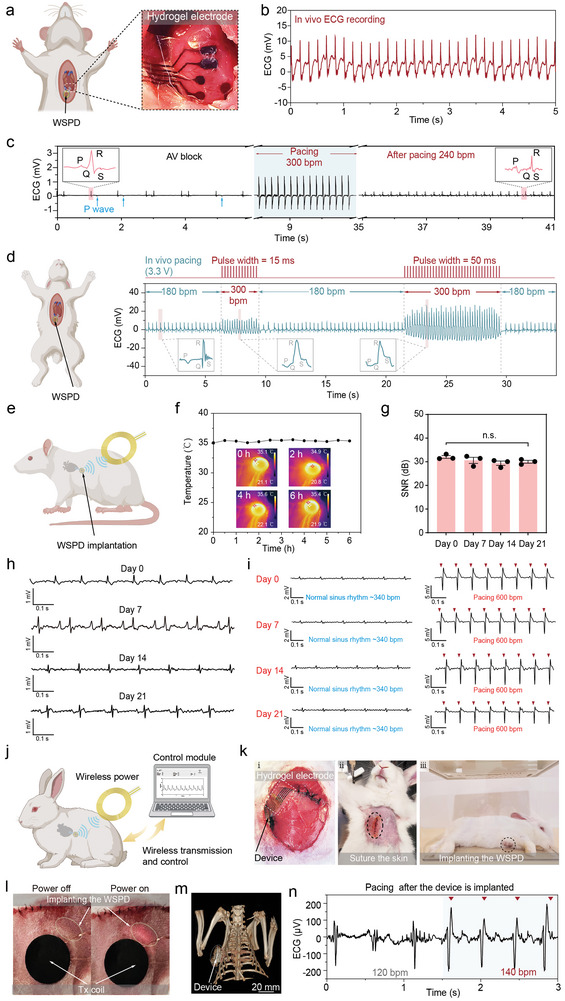
In vivo implantation of cardiac pacing. a) The schematic diagram illustrates the ECG detection and cardiac pacing of the in vivo rat heart using WSPD. The illustration shows the hydrogel electrode being applied to the surface of the heart. b) In vivo detection of rat's ECG signals by the WSPD, with a frequency of 6 Hz (*n* = 3). c) Evolution of ECG for a rate model, starting with the simulated AV block, electrical stimulation, and removal of electrical stimulation. d) Variation in rabbit sinus heart rate under stimulation with different pulse widths (5 Hz, 3.3 V) (*n* = 3). e) The schematic diagram illustrates the implantation of WSPD into the rat, with hydrogel electrodes secured at the right ventricle of the rat's heart for cardiac pacing. f) Infrared image reviewing the temperature generated by the implanted Rx coil in rats during the power supply process (*n* = 3). g) The comparison of the SNR values of the ECG signals recorded by the hydrogel electrodes on day 0, day 7, day 14, and day 21 (*n* = 3). *P* value is 0.434. h) ECG signals were detected using hydrogel electrodes on day 0, day 7, day 14, and day 21 after implantation (*n* = 3). i) ECG signals during cardiac pacing with hydrogel electrodes on day 0, day 7, day 14, and day 21 (*n* = 3). j) The illustration shows the location of the fully implanted WSPD in rabbits for cardiac pacing. k) The optical images indicate the implantation of the WSPD in the rabbit (*n* = 3). i: The hydrogel electrode is implanted into the thoracic cavity, and the wireless power supply device is implanted subcutaneously after suturing the muscle layer; ii: The rabbits that survived after skin closure; iii: After rabbit surgery, the state of awakening and recovery. l) The images depict the efficacy of powering a WSPD implanted in a rabbit using an external Tx coil. Upon receiving a stable power supply, the device activates, and its internal LED lights up, indicating its operational status (*n* = 3). m) 3D rendering of an implanted WSPD in a rabbit derived from CT images. n) ECG signals before and after pacing, recorded 6 h after implantation in rabbits (*n* = 3).

The in vivo detection and pacing functionalities of the WSPD system were further validated. Hydrogel electrodes were attached to the surface of the in vivo rat's heart and ECG signals were collected using the WSPD (**Figure** **5**a,b). To study the device's impact on ECG signals, we compared the surface ECG signals and SNR with and without WSPD‐integrated hydrogel electrode implantation in rats (Figure S30, Supporting Information). These results indicated that the materials and device did not cause noise interference in the ECG. A frequency improvement from 130 to 300 bpm in sinus rhythm was observed when electrical pulses at a frequency of 5 Hz and a voltage of 3.3 V were used for cardiac pacing (Figure S31, Supporting Information). The device was able to simultaneously collect data and pace the heart, enhancing its functionality and application range (Figure S32, Supporting Information). The atrioventricular (AV) corresponds to the interruption of impulses from the atria to the ventricles due to a functional disorder of the cardiac conduction system, typically treated with electrical pacing. We injected adenosine (AD) through the tail vein to construct the AV block model.^[^ ^15^ ^]^ After AD injection, a second‐degree AV block was detected using hydrogel electrodes.^[^ ^28^ ^]^ Electrical stimulation with hydrogel electrodes at 3.3 V and a frequency of 5 Hz restored the heart's autonomous rhythm and increased the heart rate to 240 bpm (Figure 5c). To widen the device's application, we used electrical stimulation to induce abnormal impulses in myocardial cells and abnormalities in the cardiac conduction system to model atrial fibrillation (AF). The result showed that electrical stimulation induced significant arrhythmia. Subsequently, we applied pulse stimulation using hydrogel electrodes at 3.3 V and 5 Hz (Figure S33, Supporting Information). After stimulation, the heart was restored to a normal rhythm (300 bpm). Similarly, the WSPD achieved in vivo cardiac pacing and ECG signal detection in a rabbit model (Figure 5d; Figure S34, Supporting Information). The effective induction of sinus rhythm from 180 to 300 bpm was observed during cardiac pacing. A wide pulse width corresponds to a high induced QRS waveform amplitude.

The long‐term capability of the WSPD was assessed through in vivo pacing after implantation and suturing of the surgical incision (Figure 5e). Monitoring and controlling temperature levels during the in vivo operation of the wireless pacing system is necessary to ensure animals’ health, as overheating devices can cause physiological stress, discomfort, and adverse health effects. Infrared imaging showed minor fluctuations (<0.5 °C) in the temperature rise of the device coils within 6 h of powered operation (Figure 5f), which were not significant enough to impact the animals' health. Subsequently, we investigated the long‐term feasibility of the device in vivo, spanning assessments on day 0, day 7, day 14, day 21, and day 31 (Figure 5g–i and Figure S35, Supporting Information). Post‐implantation after 21 days, the hydrogel electrode's excellent electrical performance contributed to reliable in vivo ECG recordings, with no statistically significant difference in SNR (Figures 5g, h). The application of 3.3 V pulses at 10 Hz also facilitated a sustained increase in the heart rate from 340 bpm to 600 bpm (Figures 5i). Furthermore, post‐implantation after 31 days, the hydrogel electrode still allowed for reliable in vivo ECG recordings with consistent SNR and effective cardiac pacing, increasing the heart rate from 300 bpm to 600 bpm (Figure S35, Supporting Information), demonstrating stable cardiac recording and pacing capabilities. Histological analysis indicated that the hydrogel electrode showed no significant inflammatory response on day 14 after implantation in the rat hearts (Figure S35, Supporting Information). The pacing parameter of WSPD can be adjusted by external control commands after it is implanted in rabbits (Figure 5j). The hydrogel electrodes were adhered to the right ventricular muscle to enhance pacing efficacy, and the device was implanted subcutaneously. The device was secured by sequentially suturing the rib, muscle, and skin layers (Figure 5k; Figure S36, Supporting Information). When the external power source was activated, the wireless power delivery system in the WSPD illuminated an LED, demonstrating the functionality of the bioelectronic device through the rabbit's skin (Figure 5l). Computed tomography (CT) imaging of rabbits implanted with bioelectronic devices revealed that the flexible electrode extended through the muscle and rib layers into the chest and adhered well to the surface of the heart (Figure 5m; Figures S37 and S38, Supporting Information). At 6 h post‐implantation, the application of a voltage pulse stimulus at 3.3 V from an external power supply resulted in a transition from a sinus rhythm at 120 bpm to a higher amplitude QRS wave cluster at 140 bpm, demonstrating the effectiveness of fully implantable device systems in delivering cardiac pacing therapy (Figure 5n; Movie S3, Supporting Information). These results affirmed that the WSPD, incorporated with hydrogel electrodes, allows for the effective detection of ECG signals in small and medium‐sized animals while offering reliable cardiac pacing therapy.

## 3 Conclusion

In summary, our design involves a photo‐patternable conductive hydrogel electrode characterized by outstanding biocompatibility, making it seamlessly integrated with cardiac tissues for implantation. The hydrogel electrode also showcases excellent conductivity (160 S m^−1^), stretchability (270%), flexibility (80 kPa), and stability. Its conformity to the heart's surface allows it to adapt to the dynamic environment of continuous cardiac contractions, thereby facilitating sustained long‐term (over 31 days) electrical signal conduction between the cardiac tissue and the hydrogel electrode.

Additionally, we have designed a wireless multifunctional electronic system that integrated hydrogel electrodes in a dense and flexible conjunction. In our study, we successfully demonstrated right ventricular pacing with our device effectively treats arrhythmias but still presents challenges. Future developments may focus on enhancing device performance, such as exploring the development of dual‐chamber pacing electrodes for cardiac resynchronization therapy and optimizing pacing parameters to realize the closed‐loop automatic pacing in response to the detection of arrhythmias. Moreover, we have integrated a biochemical sensor and biochemical signal detection module into the device to monitor complications arising from cardiac pacing, such as heart failure, thus enabling the further monitoring of cardiac health status. Subsequently, this device is powered through coil coupling at 220 kHz, providing sufficient power for the wireless multifunctional electronic system. This innovation offers a solution to the surgical risks associated with battery‐powered devices and infection risks arising from the mismatch between rigid electrodes and cardiac tissue. A longer biocompatibility evaluation may provide a complete insight into the device's long‐term compatibility, which will be crucial for further enhancing device design and material selection. This device presents unprecedented opportunities for implanted integrated flexible bioelectronics. It not only shows the potential for application in patients with atrioventricular block and atrial fibrillation, it can also be extended to other applications in the diagnosis and closed‐loop treatment of internal diseases, including the kidneys, liver, and intestines.

## 4 Experimental Section

### Materials

Poly(3,4‐ethylenedioxythiophene): polystyrene sulfonate (PEDOT: PSS, Clevios PH1000) was purchased from Heraeus. sulfobetaine methacrylate (SBMA), lithium phenyl (2,4,6‐trimethylbenzoyl) phosphate (LAP), 3‐(trimethoxysilyl) propyl methacrylate (TMSPMA), (3‐glycidoxypropyl) trimethoxysilane (GOPS), sodium dodecyl benzene sulfonate(DBSA), ethylene glycol (EG), bovine serum albumin (BSA), ethyl(dimethylaminopropyl) carbodiimide (EDC), mercaptoacetic acid (MA), and N‐hydroxysulfosuccinimide (NHS) were purchased from Sigma‐Aldrich. Neostigmine methanesulphate (NM) was purchased from Bide Pharmatech (Shanghai, China). N,N'‐Methylenebisacrylamide (MBAA) was purchased from Macklin Biochemical Technology (Shanghai, China). Polydimethylsiloxane (PDMS, Sylgard 184) was from Dow Corning. Bovine blood fibrinogen and thrombin were purchased from Yuanye Bio‐Technology (Shanghai, China). Ecological flexibility (Ecoflex) was purchased from Smooth‐On. PBS (pH 7.4) was purchased from Sangon Biotech (Shanghai, China). N‐terminal B‐type natriuretic peptide (NT‐proBNP) was purchased from Yibai New Biotechnology (Hangzhou, China). An anti‐NT‐proBNP antibody was purchased from Medix.

### The Fabrication of the Conductive Hydrogel Bio‐Interface Involved Several Steps

Initially, a polyethylene (PE) film was placed on a glass sheet as a substrate and thoroughly cleaned with ethanol and water, then dried with nitrogen. The substrate underwent a 5‐min oxygen plasma treatment. Following the plasma treatment, a 200 µL solution of TMSPMA was applied to the substrate, ensuring it spread evenly to cover the entire surface. This was allowed to react for 5 mins at room temperature and subsequently rinsed with deionized water before storage at a low temperature.

For the hydrogel precursor, 500 µL of PEDOT: PSS was placed in a centrifuge tube, and then 250 mg of SBMA, 5 mg of MBAA, 5 mg of LAP, and 28 mg of NM were added. The mixture was vortexed for 1 min in a low‐temperature environment. This hydrogel precursor was poured onto the substrate, forming a layer with a thickness of 100 µm, and then stored in a refrigerator at 4 °C for 24 h to achieve physical cross‐linking.

After 24 h, the hydrogel precursor was removed from the substrate and subjected to 405 nm ultraviolet light for 40 s through a photolithography plate to create the desired pattern. It was then rinsed with deionized water to remove any unexposed hydrogel, resulting in the formation of the patterned hydrogel electrode adhered to the substrate.

### Fabrication and Encapsulation of Circuit

The 4‐layer printed circuit board (PCB) designed using Altium Designer (AD) is manufactured by the commercial supplier PCBWAY. The bill of materials includes passive components such as capacitors, resistors, inductors, Schottky diodes (1N5819W, JSMSEMI), and µ‐ILED (Yongyu). Active components consist of a microcontroller unit (MCU, ESP8285, Espressif), ADC (AD7124, ADI), DAC (AD5675, ADI), signal processing chips (KS1082, Kingsense), and low dropout linear regulators (LDO, XKT3168, Xinketai). All circuit components were soldered using tin‐lead solder paste (XGSP40, melting point 183 °C, Welsolo) by hot‐air blowing.

The device encapsulation process consists of two stages. Initially, implement the parylene deposition technique to create a 30 µm parylene layer on the circuit board. This layer serves as a moisture‐resistant sealant, preventing fluid ingress and safeguarding the circuit. The silicone‐based polymer and crosslinker are thoroughly mixed in a 10:1 ratio, followed by vacuum degassing to eliminate bubbles within the PDMS. The degassed mixture is then poured into a polytetrafluoroethylene (PTFE) mold and heated at 60 °C for 2 h. Retrieve the material from the mold for the final encapsulation of the device.

### Fabrication of Flexible Interface and OECT

Polyethylene terephthalate (PET) was cleaned with ethanol and deionized water, followed by a 5‐min ion cleaning process. Subsequently, dextran was dissolved and spin‐coated onto PET at a speed of 1000 revolutions per min (rpm), followed by heating at 90 °C for 2 min. SEBS solution (13 wt% in toluene) was spin‐coated onto PET at a speed of 1,000 rpm, and then the substrate was heated at 90 °C for 4 h before further use. The photoresist (BP212‐37S) was spin‐coated on the SEBS substrate at a speed of 1000 rpm (100 °C, 2 mins) and then exposed to ultraviolet light. The photoresist pattern was formed using a developer (KMPPD238‐II), followed by heating at 90 °C for 2 min. The Cr electrode layer (12 nm) and Au electrode layer (100 nm) were deposited by thermal evaporation. Ultimately, the flexible interface based on the SEBS substrate was obtained by peeling off in isopropanol. This method was used to design electrodes of various shapes tailored to different scenarios. For example, connectors,^[^ ^26^ ^]^ Multielectrode arrays for ECG monitoring,^[^ ^29^ ^]^ needle electrodes for EEG recording,^[^ ^30^ ^]^ and electrodes for chemical signal detection.^[^ ^31^ ^]^ It can serve directly as an interconnecting layer between the hydrogel electrode and PCB, or, with a modified photolithography pattern, function as the first layer of the OECT.

After completing the first layer of the OECT, the second photolithography session prepared a PEDOT: PSS thin film pattern between the source and drain electrodes. The PEDOT: PSS dispersion (containing 5 wt % EG, 0.1 wt % DBSA, and 1 wt % GOPS) was spin‐coated on the photoresist pattern to form the channel layer of the OECT and annealed at 90 °C for 30 min. Finally, the SU‐8 photoresist was used to form an insulating layer on the Au electrodes for protection.

### Biofunctionalization of OECT

The gate electrode of the OECT was first modified with a 10 µL solution of maleic anhydride (MA) at a concentration of 1.4 mm for 7 h, followed by rinsing with deionized water. Subsequently, a mixture of equal volumes of 10 mg mL^−1^ ethyl(dimethylaminopropyl) carbodiimide (EDC) and N‐hydroxysuccinimide (NHS) solution was applied to the gate electrode for 2 h. A 5 µL solution of NT‐proBNP antibody (0.5 mg mL^−1^) was then immobilized on the gate electrode for 1 h. To prevent nonspecific binding, the gate electrodes were blocked with a 50 mg mL^−1^ solution of bovine serum albumin (BSA) for 1 h. Finally, the epidermal biosensor was rinsed and stored for further usage.

### Ex Vivo Heart Preparation

The procedure involved intraperitoneal injection of heparin (2.5 U g^−1^ diluted 1:3 with phosphate‐buffered saline) in rats for anticoagulation. After 20 min, urethane (30 mg kg^−1^ diluted 1:3 with PBS) was administered via intraperitoneal injection for anesthesia, confirmed by the cessation of pain reflex through toe pinching. The heart tissue was rapidly excised with surgical scissors and transferred to Tyrode's solution (composition: 140 mm NaCl, 5.4 mm KCl, 1 mm MgCl_2_, 1.8 mm CaCl_2_, 1.2 mm KH_2_PO_4_, 5 mm HEPES, 5.5 mm glucose). The pulmonary region was removed to expose the aorta, securely fixing it on a custom tube in Tyrode's solution to avoid air introduction. The heart was gently flushed with Tyrode's solution until no more blood was visible. Subsequently, the heart was transferred to the Langendorff system, maintained at 37 °C and oxygenated with 95% O_2_/5% CO_2_. The heart pressure was kept at 60–80 mmHg throughout the experiment. Far‐field ECG signals were recorded using Powerlab.

### Implantation of the Device in Live Rats and Rabbits

Ethical approval for animal experiments was obtained from the Science and Technology Commission of Shanghai Municipality with an accreditation number of SYXK2020‐0002.

Adult SD rats over 6 weeks old and adult New Zealand rabbits (2.5 kg) from JiHui Company were selected to implant the WSPD. Anesthesia was induced using urethane (30 mg kg^−1^ diluted 1:3 with phosphate‐buffered saline) administered via intraperitoneal injection. Subsequently, intubation was performed, and mechanical ventilation was initiated. A right thoracotomy was conducted to expose the heart, and the pericardium was excised. The hydrogel electrodes can stably adhere to the heart due to their intrinsic adhesive properties, with the electrode tips connected to a wirelessly powered device placed in subcutaneous tissue. The chest cavity, muscles, and skin were then sutured. Pacing tests were conducted by supplying power externally through a coil, placed over the skin, and recording ECG signals using Powerlab (positive electrode on the right foreleg, negative electrode on the left foreleg, ground electrode on the hind leg).

### Cell and Tissue Biocompatibility for Hydrogel

Ethical approval for animal experiments was obtained from the Science and Technology Commission of Shanghai Municipality with an accreditation number of SYXK2020‐0002.

Human embryonic kidney 293 (HEK293) cells and cardiomyocytes were chosen for in vitro biocompatibility assessment. We specifically extracted cardiomyocytes from neonatal mice to verify the biocompatibility of the hydrogel electrodes. Neonatal mice aged 1–4 days are obtained from JiHui Company, and their skin is disinfected with 75% ethanol. The heads and limbs of the neonates are secured with pins. The skin of the chest is incised, and the subcutaneous tissue is disinfected with 75% ethanol. A fresh pair of forceps and scissors are used to open the thoracic cavity, and the heart is extracted. The heart is placed in a dish (or penicillin bottle) containing D‐Hanks solution. The atria are removed and the ventricles are dissected. The ventricles are rinsed three times with D‐Hanks solution to remove any remaining blood. The heart is cut into fragments ≈1 mm^3^ in size and transferred to a centrifuge tube. 5 mL of digestive solution is added and the mixture is incubated at 37 °C for 5 min. The fragments are allowed to settle naturally and the supernatant is discarded. An additional 5 mL of digestive solution is added, and digestion is continued at 37 °C for 20 min, shaking every 2 min. After digestion, the solution is pipetted for 1 min and any undigested heart fragments are transferred to another centrifuge tube. 2 mL of cold culture medium is added to stop the digestion, and the mixture is centrifuged at 1000 rpm for 5 min. The supernatant is discarded and 2 mL of D‐Hanks solution is added to the precipitate. The mixture is centrifuged at 1500 rpm for 10 min. The supernatant is discarded and 2 mL of culture medium is added to the precipitate. The solution is pipetted to create a cell suspension. The undigested heart fragments are further digested with an additional 5 mL of digestive solution and the steps above are repeated. The cell suspensions are combined and placed in a culture flask, which is then Incubated in a CO_2_ incubator at 37 °C with 5% CO_2._

The cell culture medium consisted of 10 mL of DMEM supplemented with 10% v/v fetal bovine serum and 100 U mL^−1^ penicillin‐streptomycin. After being sterilized by UV irradiation for 12 h, 10 mg of hydrogel was directly added to a 10 mL culture medium for 48 h. Subsequently, the hydrogel was removed, cells were cultured in a medium containing hydrogel's extracts, and the co‐culture was maintained for 24 h at 37 °C in a 5% CO_2_ environment.^[^ ^7b^ ^]^ Cell viability was assessed using the Live/Dead assay (Aladdin). Live and dead cells were labeled with Calcein‐AM (2 µm in PBS, green, ex/em = 490/515 nm) and propidium iodide (PI, 4.5 µm in PBS, red, ex/em = 535/617 nm) by staining in darkness for 15 min in PBS, respectively. Fluorescence images were captured using a Nikon laser scanning confocal microscope (Nikon, Japan) for subsequent statistical analysis.

Hydrogel and Pt electrodes were injected into the subcutaneous tissue of the backs of rats for 7 days, respectively. The collected skin tissue was fixed using paraformaldehyde (4 wt%) for 48 h at room temperature. Histological analysis was performed by H&E staining.

### Characterization of the Interface Between Hydrogel Electrode and Heart

The interface between the hydrogel and heart tissue was analyzed using confocal microscopy and scanning electron microscopy (SEM). The hydrogel labeled with red‐MA fluorescence, was affixed to the heart tissue using fibrin glue. Subsequently, the heart tissue was excised and fixed with 4% paraformaldehyde (PFA) at 4 °C for 48 h. After fixation, the heart tissue underwent dehydration with 10% and 30% sucrose for 48 h each at 4 °C. The tissue was further fixed with an embedding agent and frozen and sectioned into 100 µm slices. These slices were treated with 0.3% Triton X‐100 for 3 min followed by staining with Alexa Fluor 488 Phalloidin (66 µm, 1:40) and DAPI (5 mm, 1:100) for 20 min at room temperature. Confocal microscopy was employed to characterize the interface between the hydrogel and heart tissue.

For SEM analysis, the heart tissue was fixed with 2.5% glutaraldehyde in PBS for 1 h at room temperature, followed by overnight storage at 4 °C. After rinsing with PBS three times, the samples underwent dehydration using a gradient ethanol series (10%, 30%, 50%, 70%, 100%), and then start‐butanol. Finally, the samples were dried in a freeze dryer and sputter‐coated with gold for SEM observation.

### Material and Chemical Characterizations

FTIR measurements were performed using the Thermo Scientific Nicolet iS20 instrument from the United States, with spectra recorded over the range of 600–4000 cm^−1^ at a resolution of 4 cm^−1^ and 32 scans. Raman spectroscopy was conducted with the Horiba LabRAM HR Evolution instrument, employing a laser wavelength of 633 nm, and spectra were acquired over the range of 1000–1600 cm^−1^. Small‐angle X‐ray scattering (SAXS) analysis was carried out using the Xenocs Xeuss 3.0 instrument from France. X‐ray photoelectron spectroscopy (XPS) measurements were conducted using the Thermo Scientific K‐Alpha instrument from the United States, with a spot size of 400 µm, and an operating voltage of 12 kV. The CT imaging of devices implanted in the rabbit using uCT528 (Shanghai United Imaging Healthcare) involves the utilization of advanced imaging technology provided by the specified equipment.

The electrochemical properties of the hydrogel were investigated using a CHI660e electrochemical workstation (CH Instruments, China). A three‐electrode system was employed with the hydrogel as the working electrode, an Ag/AgCl electrode as the reference electrode, and a Pt as the counter electrode. PBS with a concentration of 0.1 m at a pH of 7 was utilized as the electrolyte. EIS was conducted over a frequency range of 1 Hz to 100 kHz. CV curves were recorded within a potential window of 0.2 to 0.8 V at a scan rate of 20 mV s^−1^. Additionally, a Controlled‐Current Chronoamperometry (CIC) technique was applied with a voltage of ±0.5 V and a pulse width of 10 ms. The OECT was measured using the Keithley 2600 digital source meter from Tektronix.

### Statistical Analysis

Data are presented as mean ± standard deviation (SD) or standard error of the mean (SEM) and were analyzed with Origin 9.0 and GraphPad Prism software. Statistical analysis was performed using an unpaired student's *t*‐test. *p < 0.05, **p < 0.01, ***p < 0.001, and ****p < 0.0001.

## Conflict of Interest

The authors declare no conflict of interest.

## Author Contributions

Z.C. and B.W. contributed equally to the manuscript. Y.F. conceived the project and devised the experimental plan. Z.C. and B.W. synthesized materials and conducted material characterizations. Z.C. and B.G. were involved in device design and testing, as well as the development of the wireless control platform. Z.C., B.W., and Y.L. designed and prepared electrochemical sensors, performing relevant experiments. Z.C., Q.R. and M.R. focused on animal experiments. Z.C., B.W., Q.R., J.N., Y.L, D.J., M.R., and Y.Z. collected and analyzed the data. Y.F., Z.C., and B.W. collaborated on manuscript writing. All authors engaged in result discussions and contributed to the manuscript.

## Supporting information

Supporting Information

Supplemental Movie 1

Supplemental Movie 2

Supplemental Movie 3

## Data Availability

Research data are not shared.
