# Supplementary material for: Fully Implantable Wireless Cardiac Pacing and Sensing System Integrated with Hydrogel Electrodes
Source: Adv Sci (Weinh). 2024 Sep 29;11(44):2401982. doi: 10.1002/advs.202401982 (PMC11600267; doi:10.1002/advs.202401982)
Supplement: Supplementary file 1 — Supporting Information [file ADVS-11-2401982-s003.docx]

Supporting Information

**Fully Implantable Wireless Cardiac Pacing and Sensing System Integrated with Hydrogel Electrodes**

*Zhiqiang Chang ^a #^, Bingfang Wang ^a #^, Qinjuan Ren ^a^, Jianfang Nie ^a^, Bihan Guo ^a^, Yuhan Lu ^a^, Xinxin Lu ^a^, Ya Zhang ^a^, Daizong Ji ^a^, Yingying Lv ^b^, Menahem Y. Rotenberg ^c^, Yin Fang ^a^ **

^a^ Research Center for Translational Medicine, Medical Innovation Center and State Key Laboratory of Cardiology, Shanghai East Hospital; The Institute for Biomedical Engineering & Nano Science, Tongji University School of Medicine, Shanghai, 200120, China

^b^ Research Centre of Nanoscience and Nanotechnology, College of Science, Shanghai University, Shanghai 200444, China

^c^ Department of Biomedical Engineering, Technion-Israel Institute of Technology, Haifa, Israel

^#^ The authors contributed equally to the manuscript.

* Correspondence: E-mail: [yin_fang@tongji.edu.cn](mailto:yin_fang@tongji.edu.cn).

This file includes:

Figures S1 to 38, Table S1 to S3

Other Supplementary Material for this manuscript includes the following:

*Movies S1 and S3*


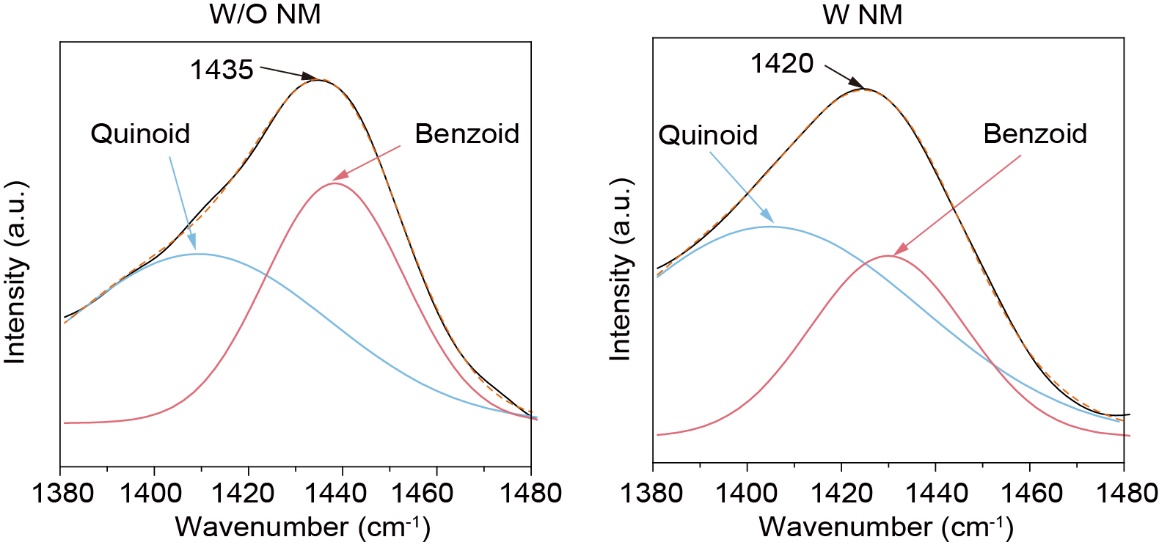


**Figure S1.** Raman spectroscopy peak fitting curves. Images demonstrating a transformation from the benzoid (1436 cm^−1^) to quinoid (1410 cm^−1^) structure to fit the Raman spectra of hydrogels with and without NM.


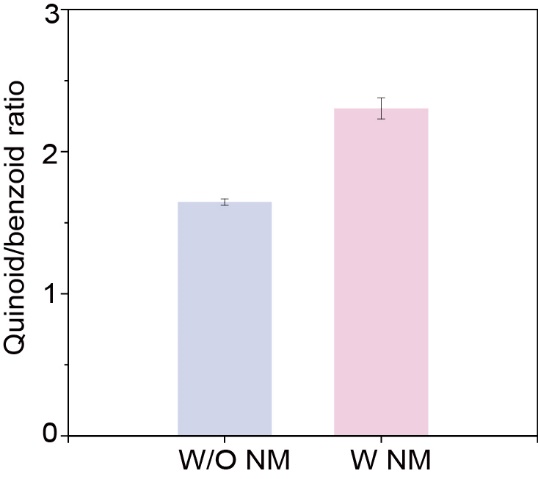


**Figure S2.** Raman spectrum analysis for the hydrogels with and without NM. The addition of NM increased the ratio of quinoid structure to benzoid structure in the hydrogels (*n*=3).


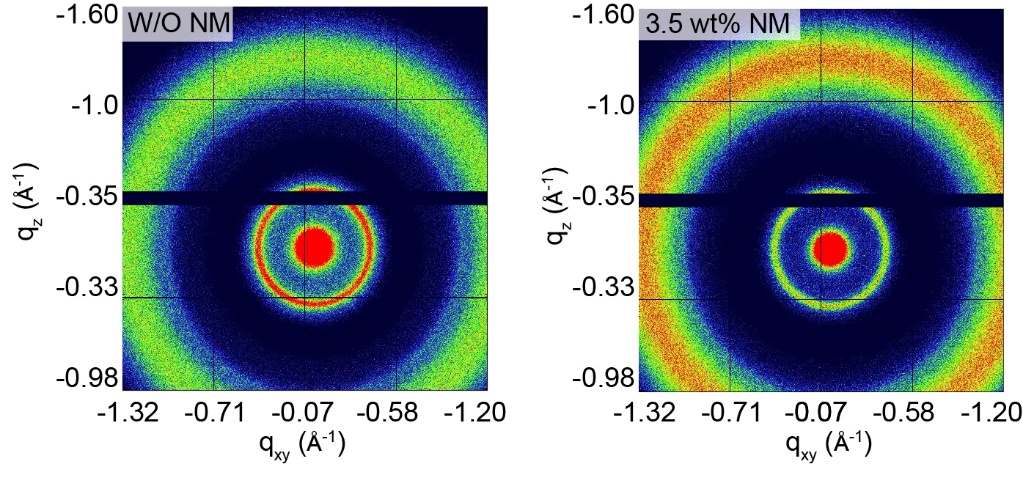


**Figure S3.** Two-dimensional SAXS images. Two-dimensional SAXS images vividly illustrate changes in the internal crystallinity of PEDOT of the hydrogels with and without NM.


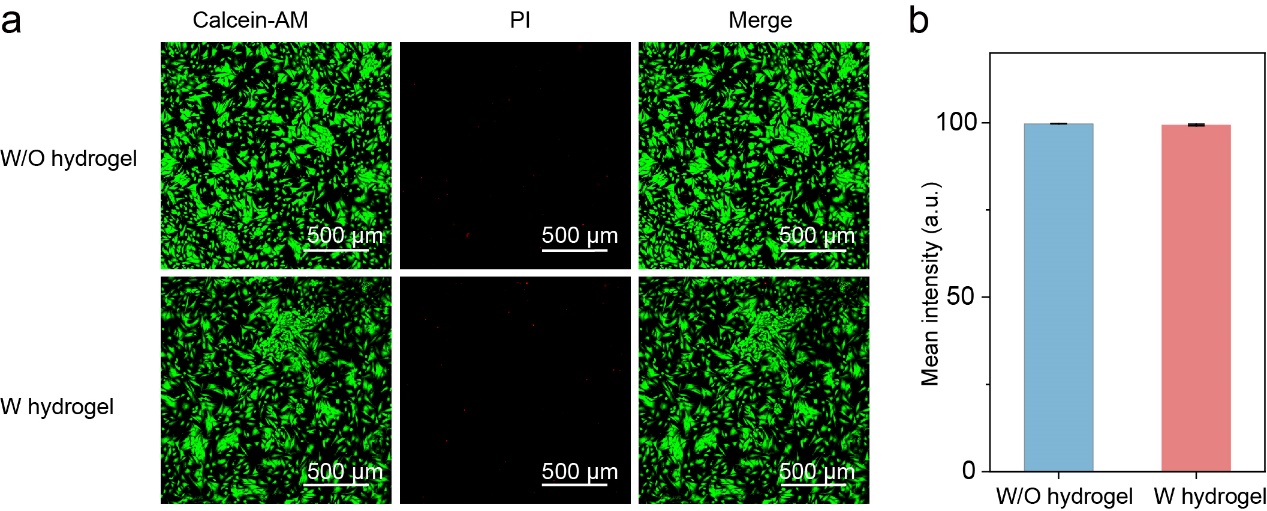


**Figure S4.** Hydrogel biocompatibility of HEK293 cells. a) Confocal images of HEK293 cells cultured in a medium containing hydrogel extracts. b) Viability and death statistics of HEK293 cells (*n*=3).


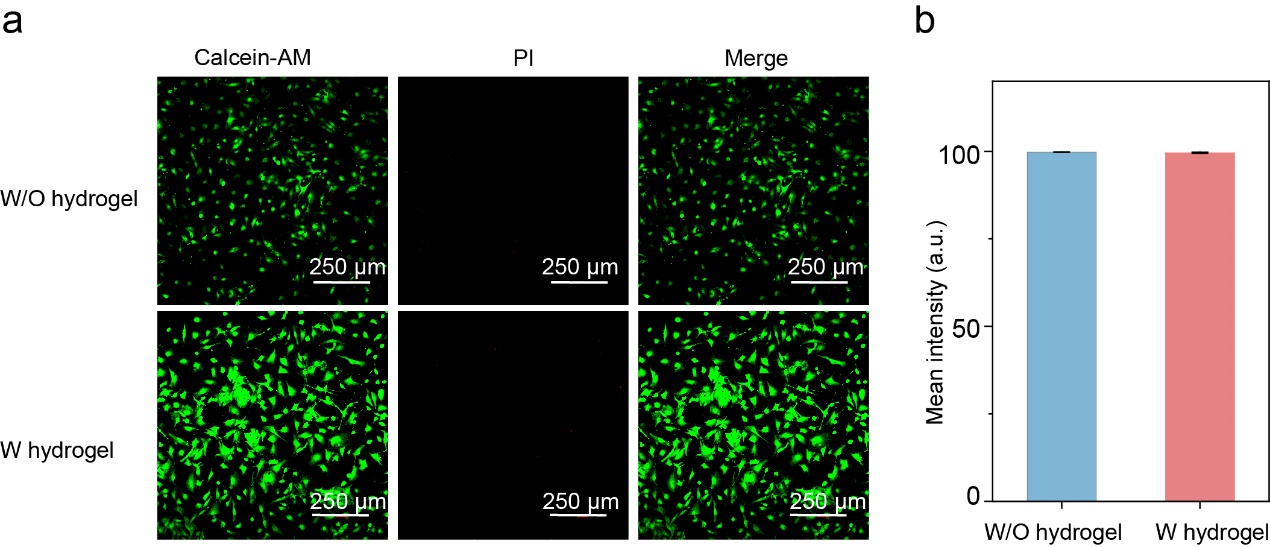


**Figure S5.** Hydrogel biocompatibility of cardiomyocytes. a) Confocal images of cardiomyocytes cultured in a medium containing hydrogel extracts. b) Viability and death statistics of cardiomyocytes (*n*=3).


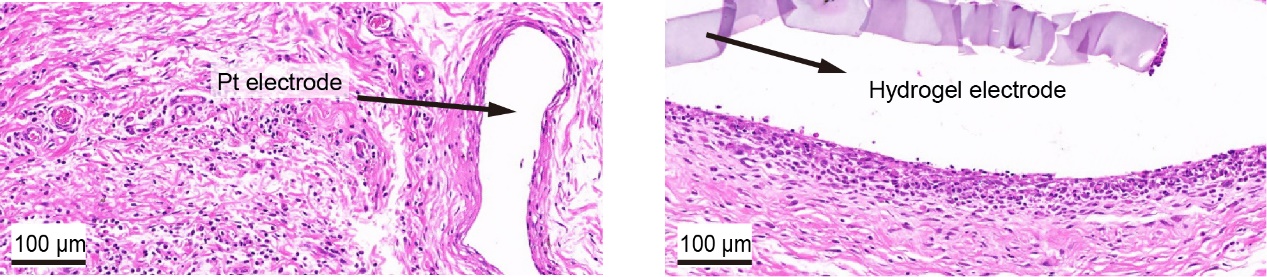


**Figure S6.** H&E staining of rat skin tissue. Images of the subcutaneous implantation of the hydrogel and Pt electrode in the subcutaneous tissue of the backs of rats for 7 days (*n*=3).


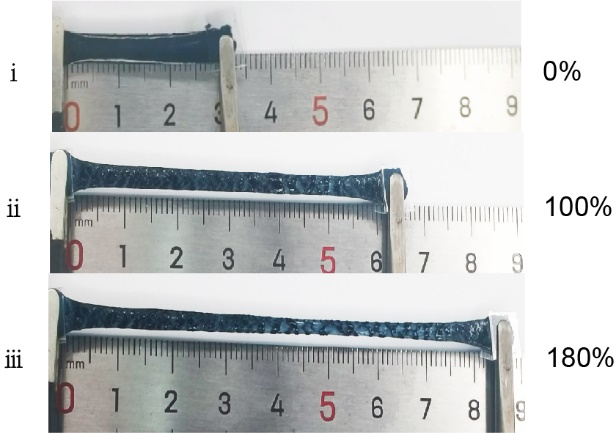


**Figure S7.** Optical image of the stretched hydrogel. Photographs depicting the stretching of the hydrogel and maximum tensile strain of 200%, with dimensions of 1 mm×5 mm×30 mm.


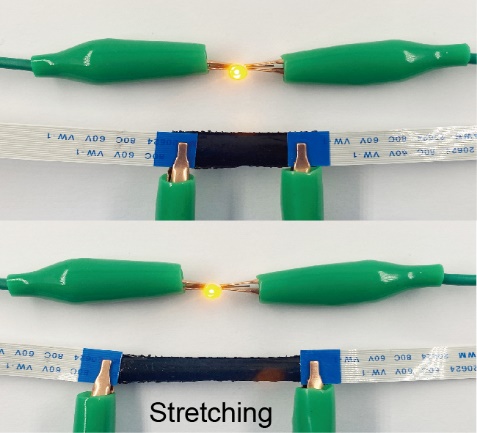


**Figure S8.** The conductive effects under stretching of the hydrogel. The hydrogel serves as a conductor to illuminate a light-emitting diode (LED), and the brightness remains before and after stretching.


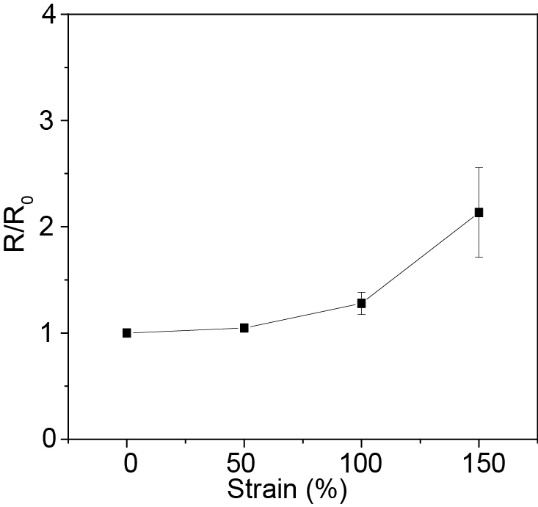


**Figure S9.** The resistance changes of the hydrogel under stretching. The hydrogel remained insensitive to strain at and below 100% (*n*=3).

**
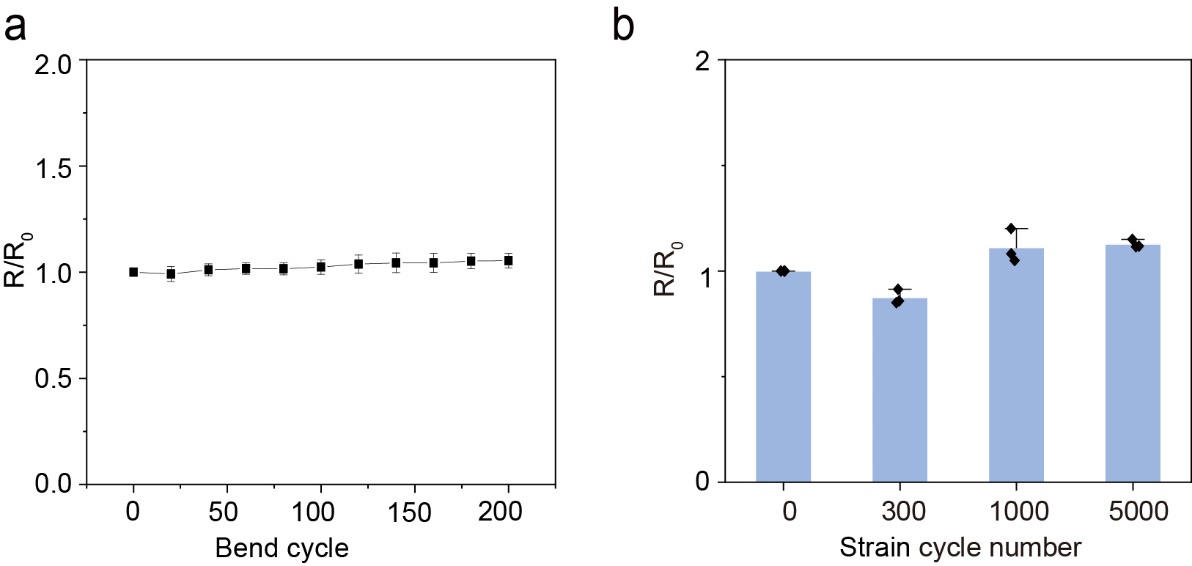
**

**Figure S10.** Resistance variation of the hydrogel under cyclic stretching. After 5000 cycles of 35% tensile strain, the resistance of the hydrogel shows minimal variation (*n*=3).


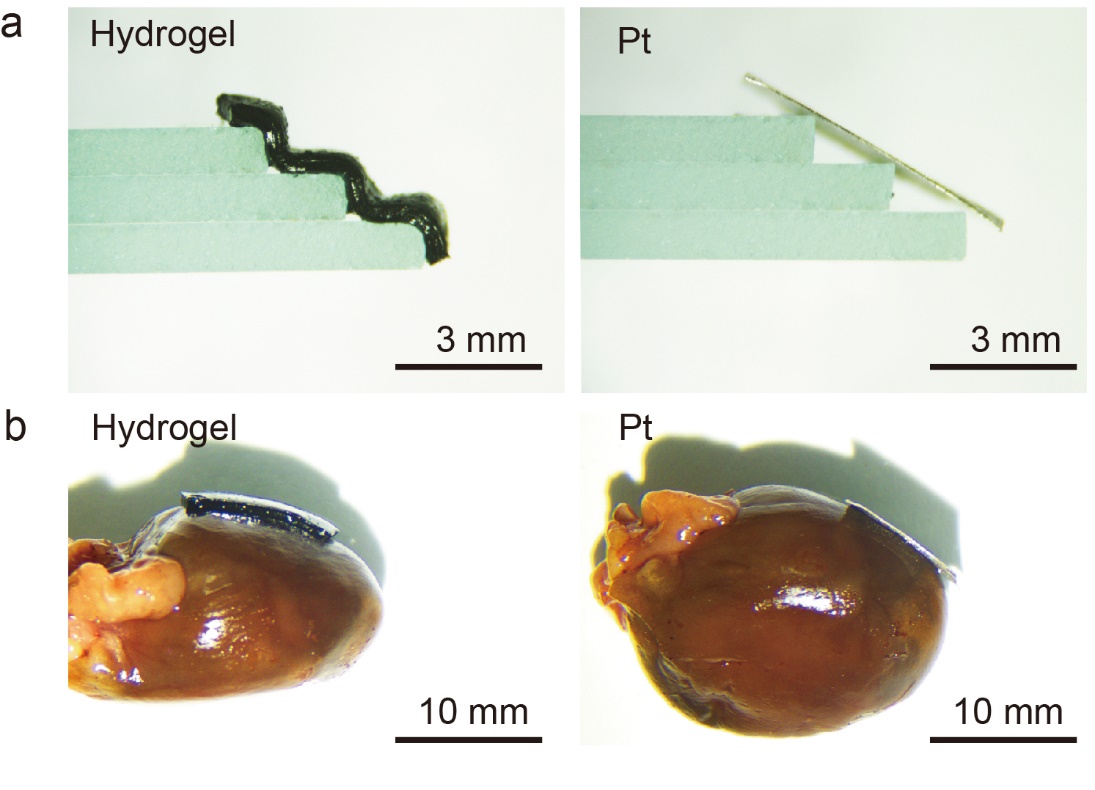


**Figure S11.** a) Hydrogel and Pt film on right angle step for exhibition of curviness. b) Hydrogel and Pt thin films on the curved surface of the heart (*n*=3).


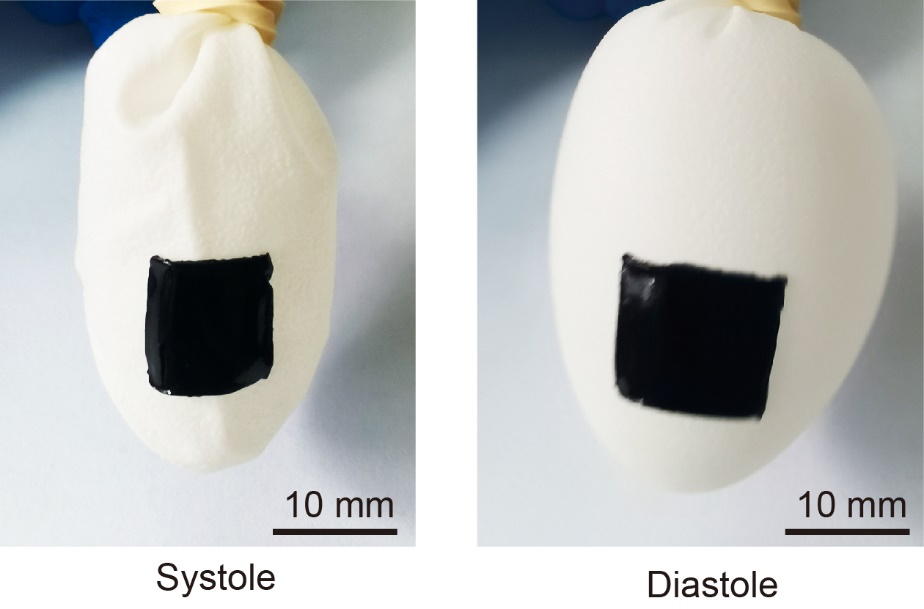


**Figure S12.** Deformation of the hydrogel electrode in response to the systole and diastole process.


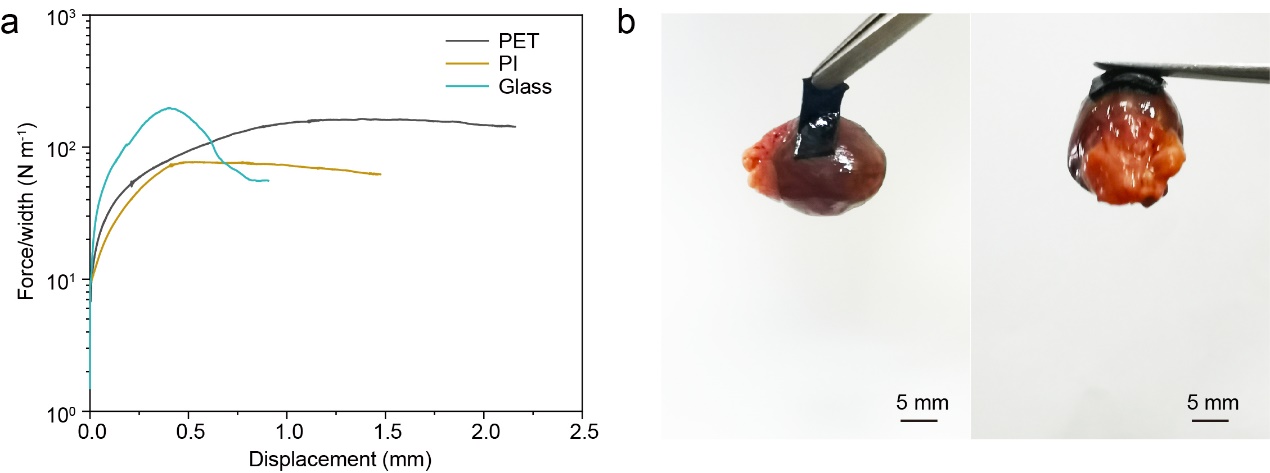


**Figure S13.** a) Representative curves of the peeling force per width (F/W) versus displacement for the hydrogel coating on the PET, PI, and glass substrate. b) The hydrogel electrode adheres to the rat's heart (*n*=3).


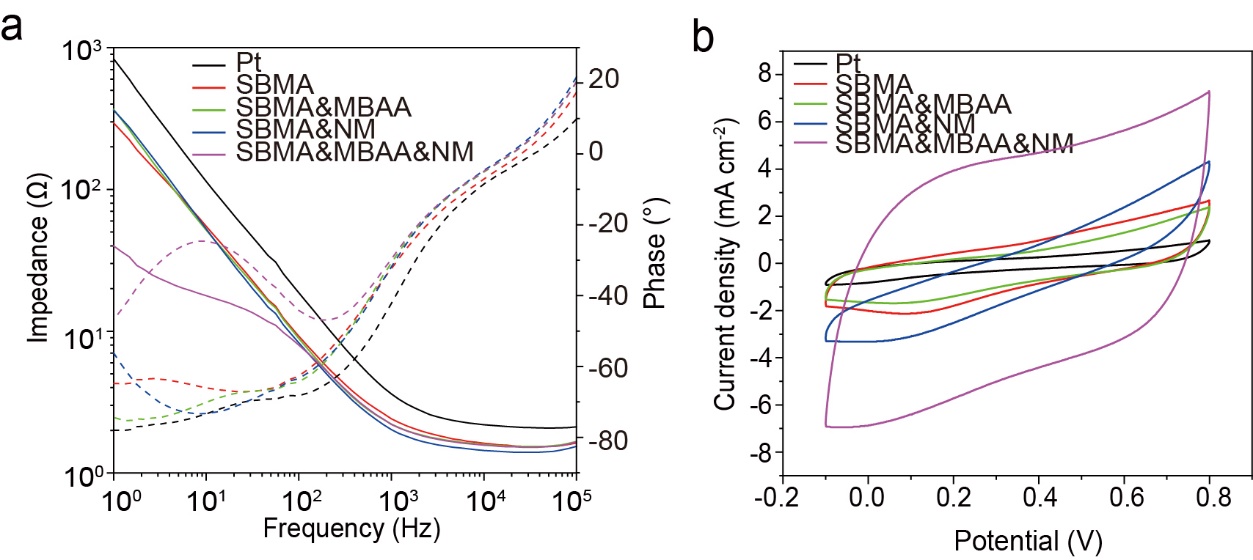


**Figure S14.** EIS and CV curves of hydrogels with different compositions compared to a Pt electrode. a) EIS of the various hydrogels, with an area of 0.3 cm^2^ and a frequency range of 1−10^5^ Hz. solid line for impedance and dashed line for phase b) CV curves illustrating Pt electrodes with various hydrogel composition.


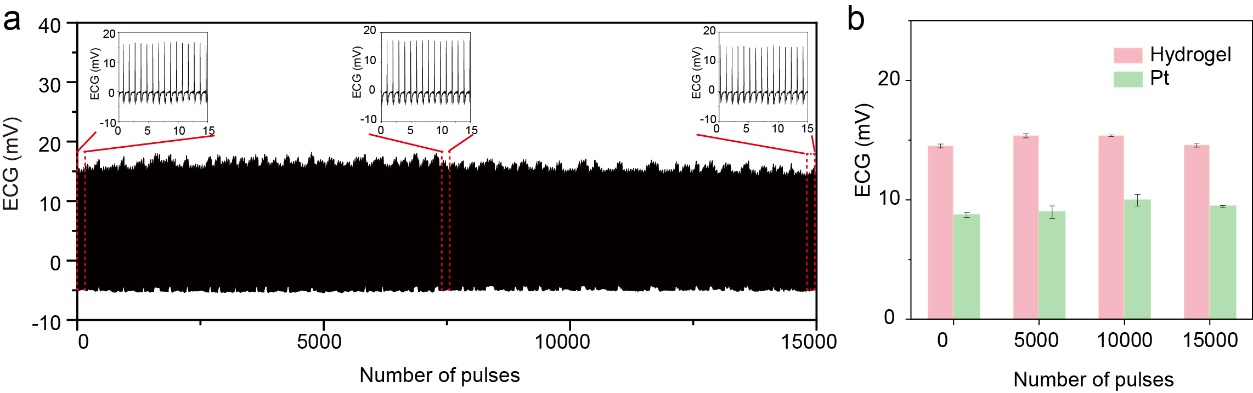


**Figure S15.** a) Long-term ECG signals in vivo cardiac pacing with 3.3 V, 5 Hz pulses over 15000 cycles by hydrogel electrode (*n*=3). b) Amplitude of the ECG signals generated by Pt and hydrogel electrodes during stimulation over 15000 cycles (*n*=3).


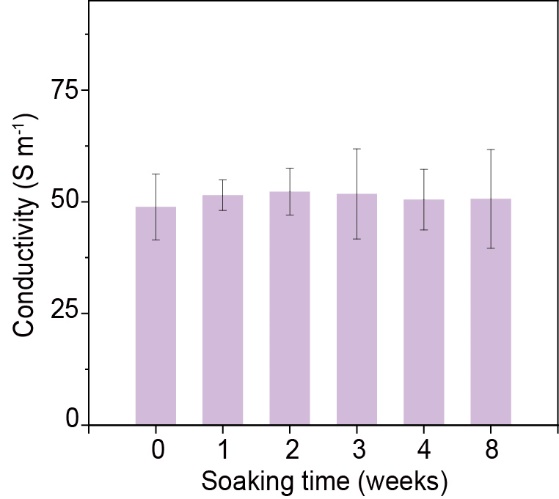


**Figure S16.** The variation in the conductivity of the hydrogels in water. The conductivity variations of the hydrogels showed minimal variation after being immersed in water for 8 weeks (n=5).


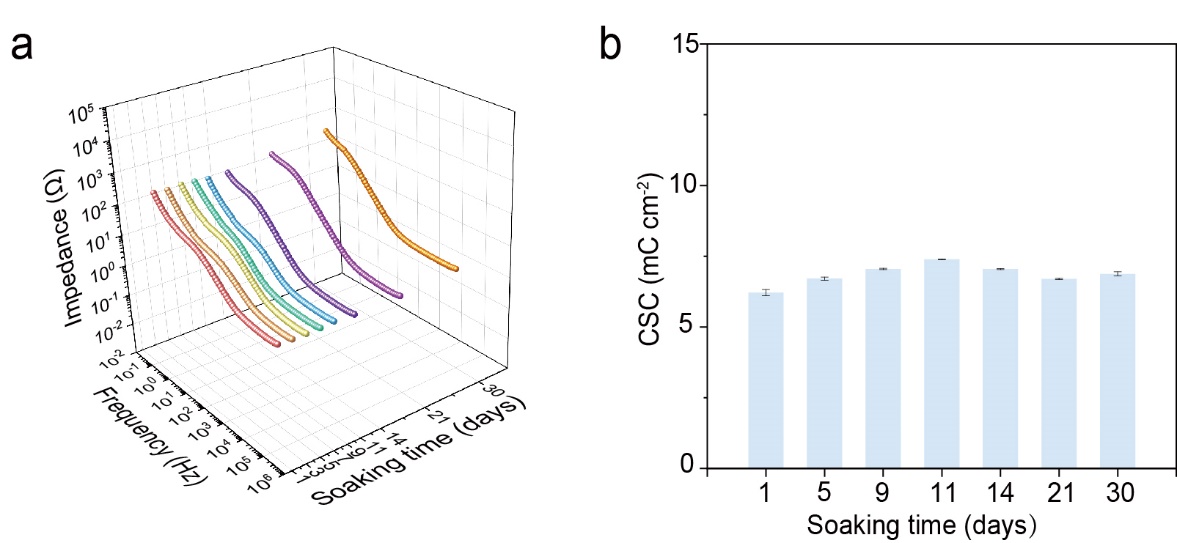


**Figure S17.** The electrochemical stability of the hydrogels in PBS. a) EIS curves and b) CSC of the hydrogels demonstrate negligible fluctuations in PBS over 30 days (*n*=3).


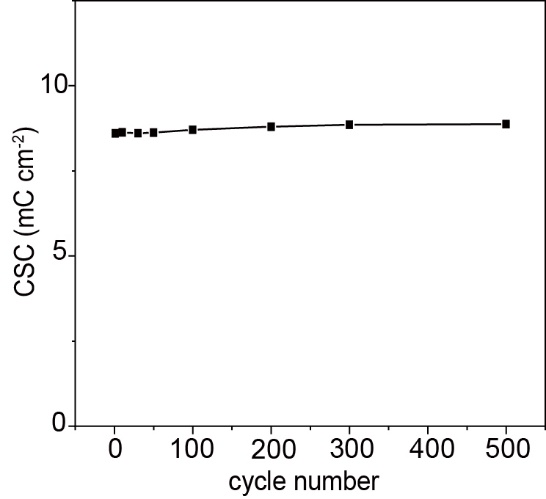


**Figure S18.** Long-term operational stability of the hydrogels. Hydrogels exhibit marginal changes in their CSC for 500 cycles.


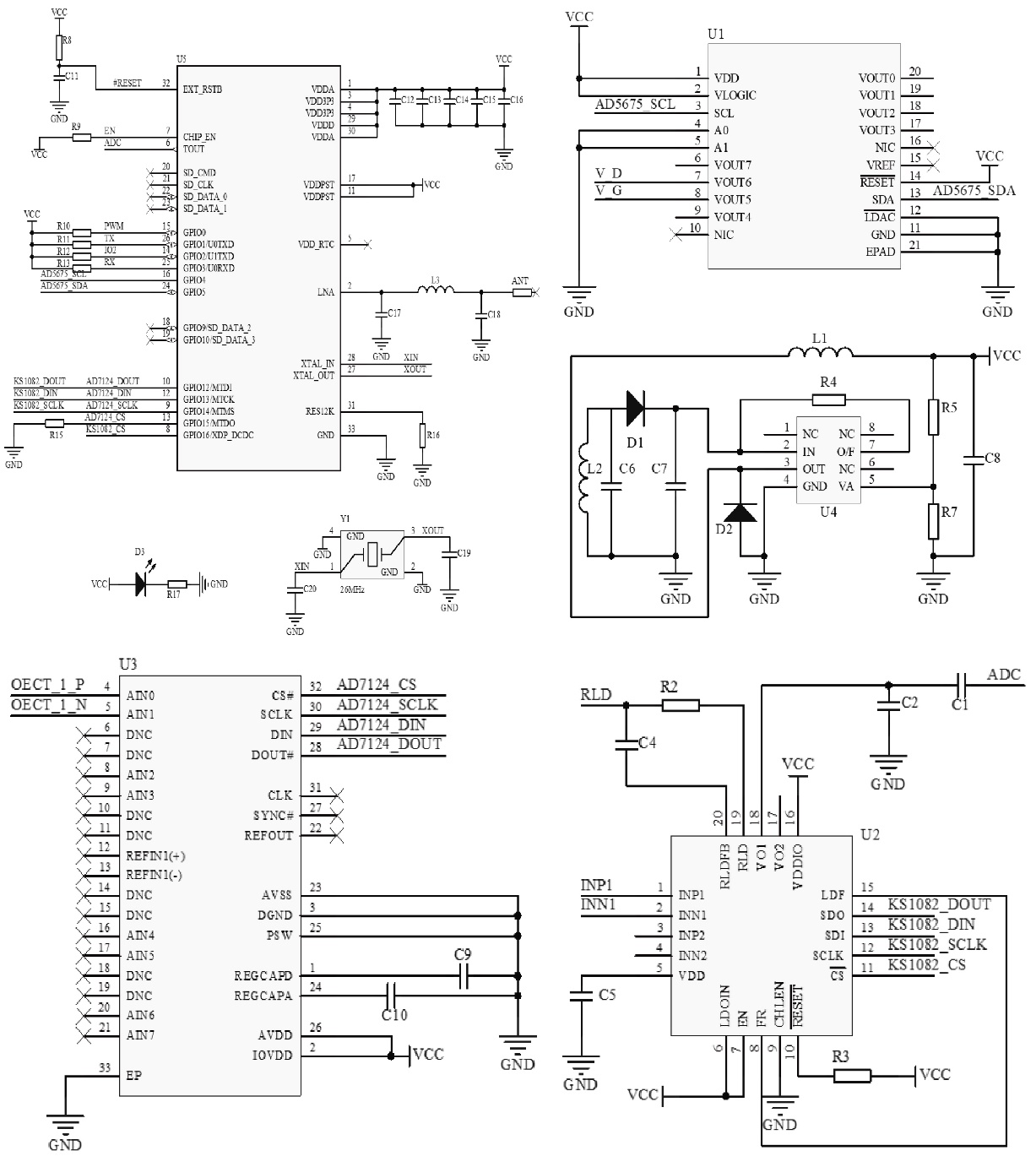


**Figure S19.** The rectifier circuit converts the inductively coupled signal to an approximate DC output, and the low-voltage linear regulator T3168 stabilizes the voltage at 3.3 V to power the device. The ESP8285 microcontroller communicates with the AD5675 via the I2C interface to drive the electrochemical sensor and with the KS1082 ECG signal detection chip via the SPI interface to control ECG signal processing parameters. The ECG signals are received by the internal ADC of the ESP8285 and converted into digital signals. Using the internal SPI interface's time-division multiplexing feature, the ESP8285 communicates with the AD7124 to adjust electrochemical signal processing parameters and receive data. The ESP8285 generates adjustable pulse signals for cardiac pacing stimulation via its internal DAC. When the power supply is low, the ESP8285 enters a sleep state, and the pulse stimulation output pin directly connects to the LDO output pin, allowing external devices to provide stimulation pulses for basic cardiac pacing functions. Signals detected by the ESP8285 are transmitted to an upper computer for data display and storage via a 2.4 GHz antenna.


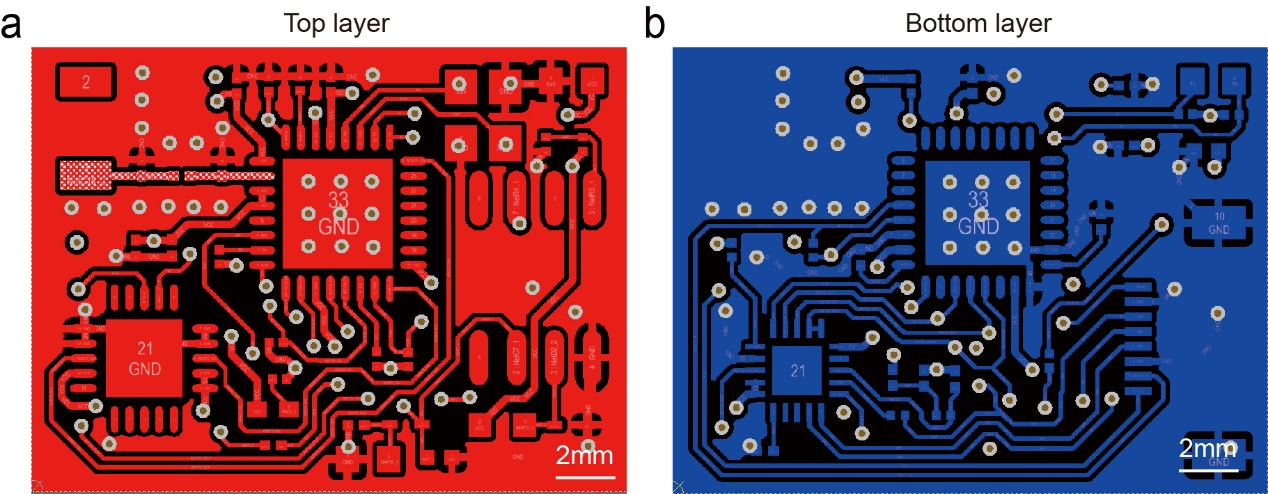


**Figure S20.** Multilayer structure of a PCB. a) Top layer circuit layout of the PCB. b) The bottom layer circuit layout of the PCB.


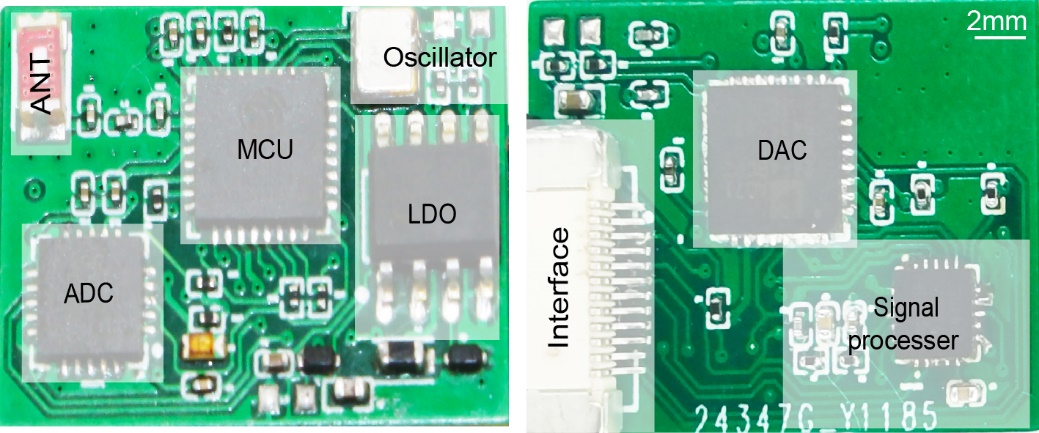


**Figure S21.** Photograph of the bioelectronic device. Left: The top layer of the bioelectronic device contains an antenna, an MCU, an LDO, and an ADC chip. Right: The bottom layer of the bioelectronic device includes components such as the signal processing chip, the DAC chip, and the bioelectronic interface.


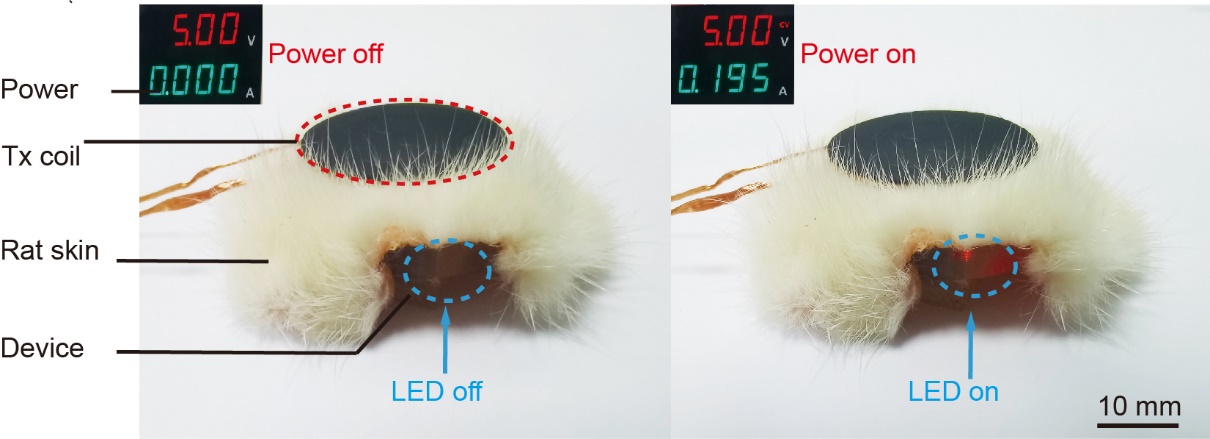


**Figure S22.** Wireless power transmission. Pictures showing an external radio frequency (RF) coil powers the WSPD through the skin. The external power supply is activated, causing the transmitting coil to emit an RF signal resonantly coupled between the coils. The receiving coil generates an alternating current signal, rectified and regulated to provide power to the device, with the LED indicating activation (*n*=3).


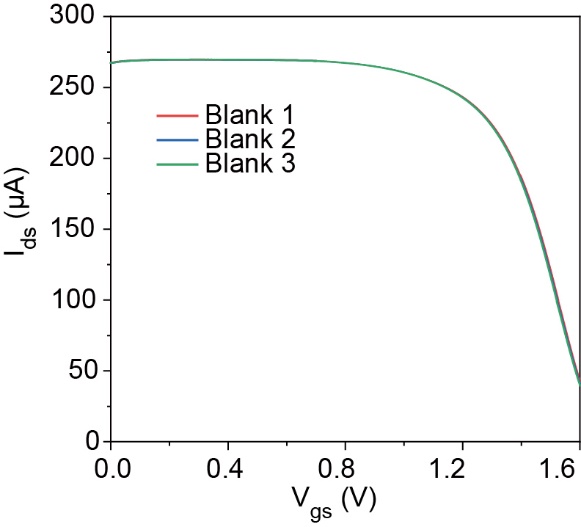


**Figure S23.** Transfer curves of the OECT. The repeatability of the OECT was verified, with the curves showing almost no deviation.


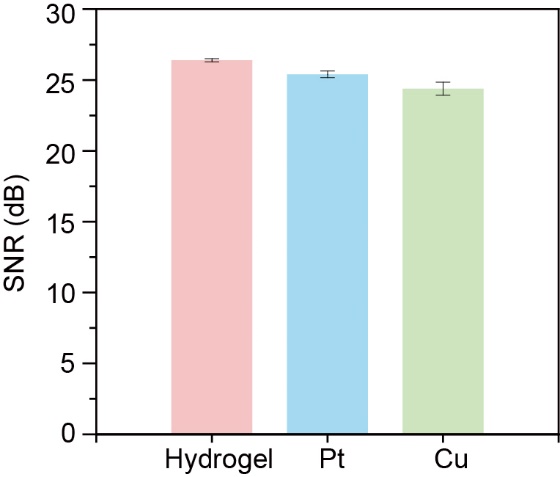


**Figure S24.** SNR assessment in the detection of ECG signals. The SNR of ECG signals detected by hydrogel electrodes is higher compared to Pt electrodes and copper electrodes (*n*=3).


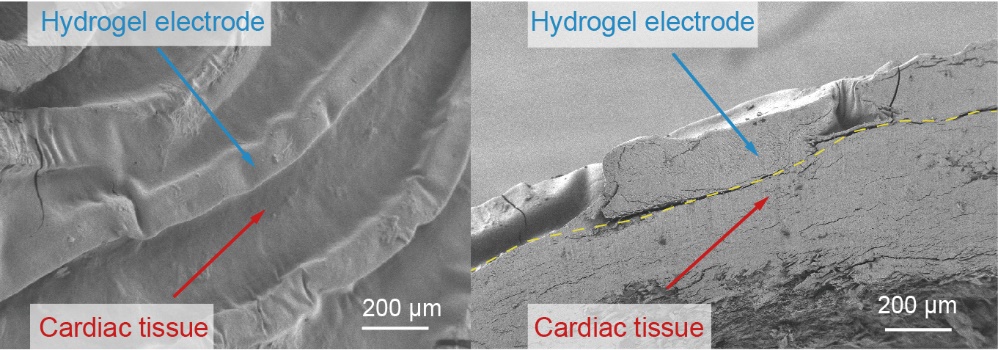


**Figure S25.** SEM images of the hydrogel electrode-cardiac tissue interface. SEM images reveal a robust attachment of the hydrogel electrode to the cardiac tissue, indicating a secure and firm connection (*n*=3).


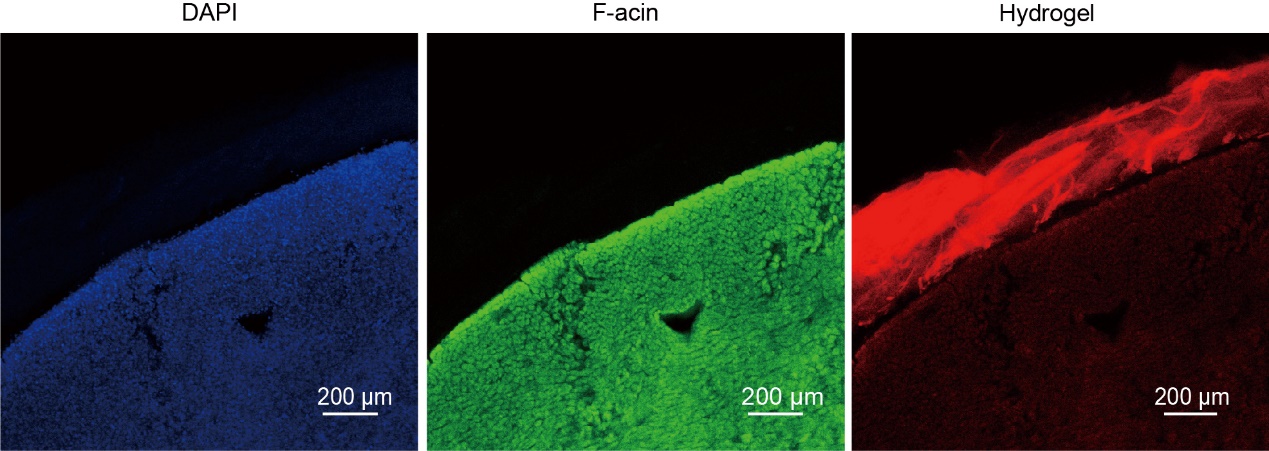


**Figure S26.** The confocal images of the interface between cardiac tissue and the hydrogel electrode. The confocal images of the interface between cardiac tissue and the hydrogel electrode demonstrate the hydrogel's excellent adhesive capability relative to the cardiac surface.


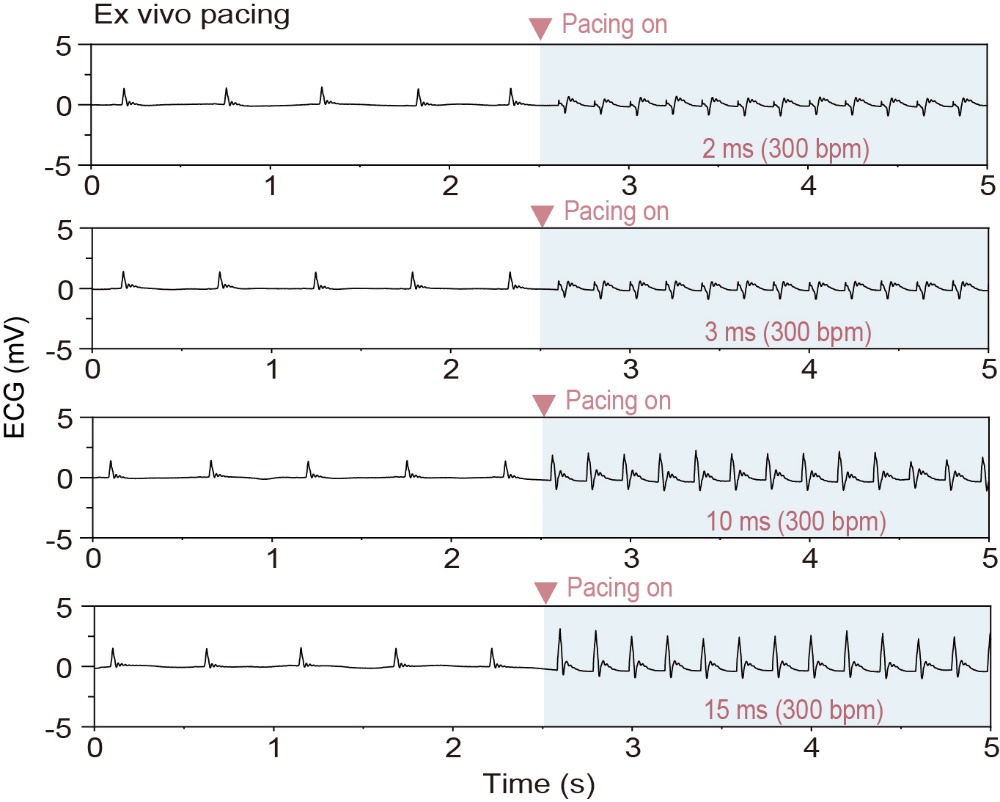


**Figure S27.** ECG signals before and during pacing. Sinus rhythm changes in the heart are induced by different pulse widths, with a voltage of 3.3 V and a frequency of 5 Hz.


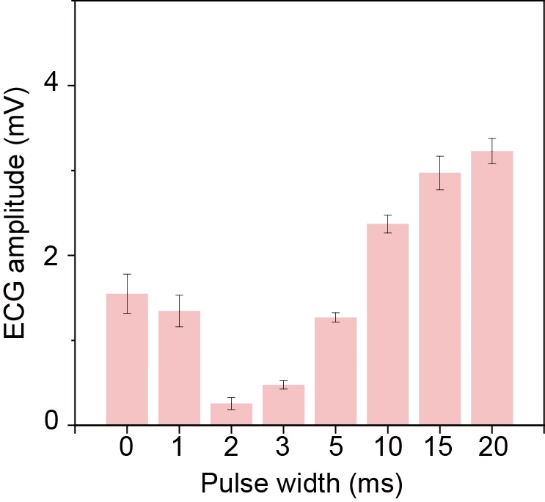


**Figure S28.** Amplitude of ECG signals. Effect of different pulse widths on the ECG amplitude, with a voltage of 3.3 V, and a frequency of 5 Hz (*n*=3).


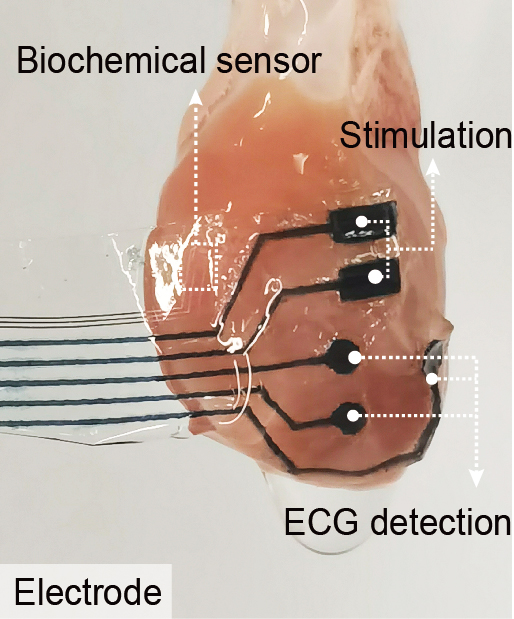


**Figure S29.** Hydrogel electrode on ex vivo heart perfusion. OECT is a biochemical sensor that detects abnormal NT-proBNP concentrations in the ex vivo heart (*n*=3).


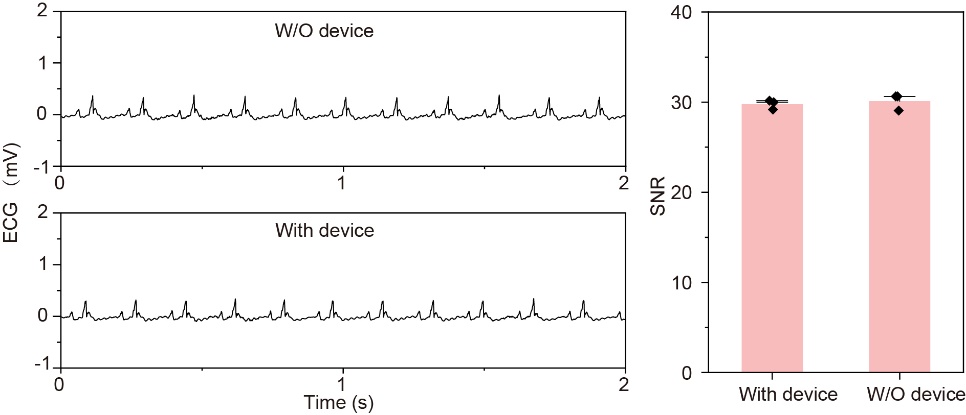


**Figure S30.** The ECG and SNR before and after implanting the device into the heart (*n*=3).


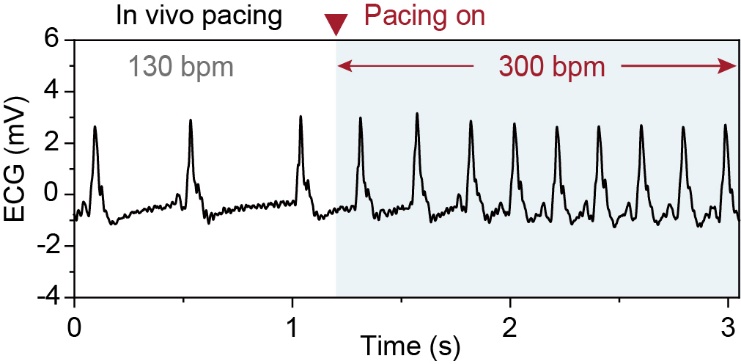


**Figure S31.** In vivo ECG signals in rats. Changes in cardiac rhythm induced by the voltage pulse stimulation (5 Hz, 3.3 V) on the beating heart of the rat (*n*=3).


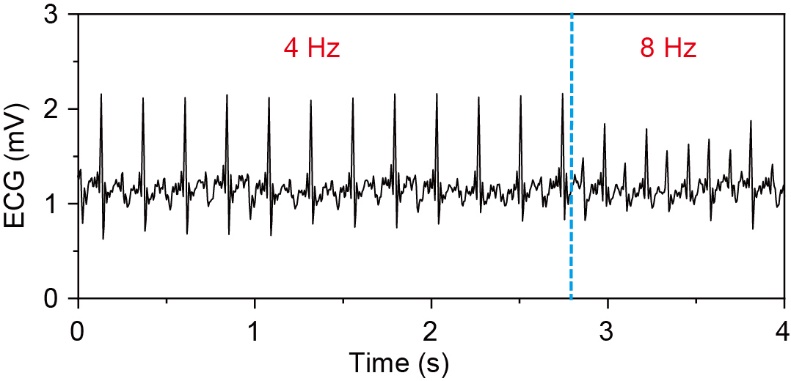


**Figure S32.** The ECG signals are recorded simultaneously during cardiac pacing with an implanted WSPD (*n*=3).


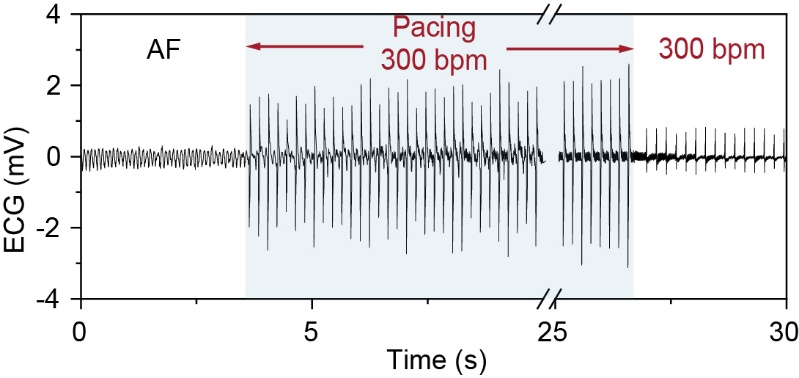


**Figure S33.** Hydrogel electrode stimulation for atrial fibrillation treatment includes the detection of atrial fibrillation signals, the process of electrical stimulation, and the restoration of normal sinus rhythm after stimulation (*n*=3).


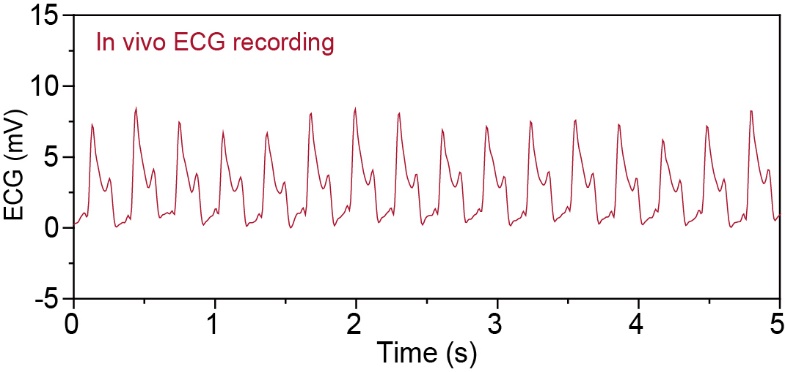


**Figure S34.** ECG signals of rabbits were recorded by the WSPD implanted in the rabbit. The implementation of WSPD detects rabbit ECG signals with a frequency of 180 bpm and an amplitude close to 8 mV (*n*=3).


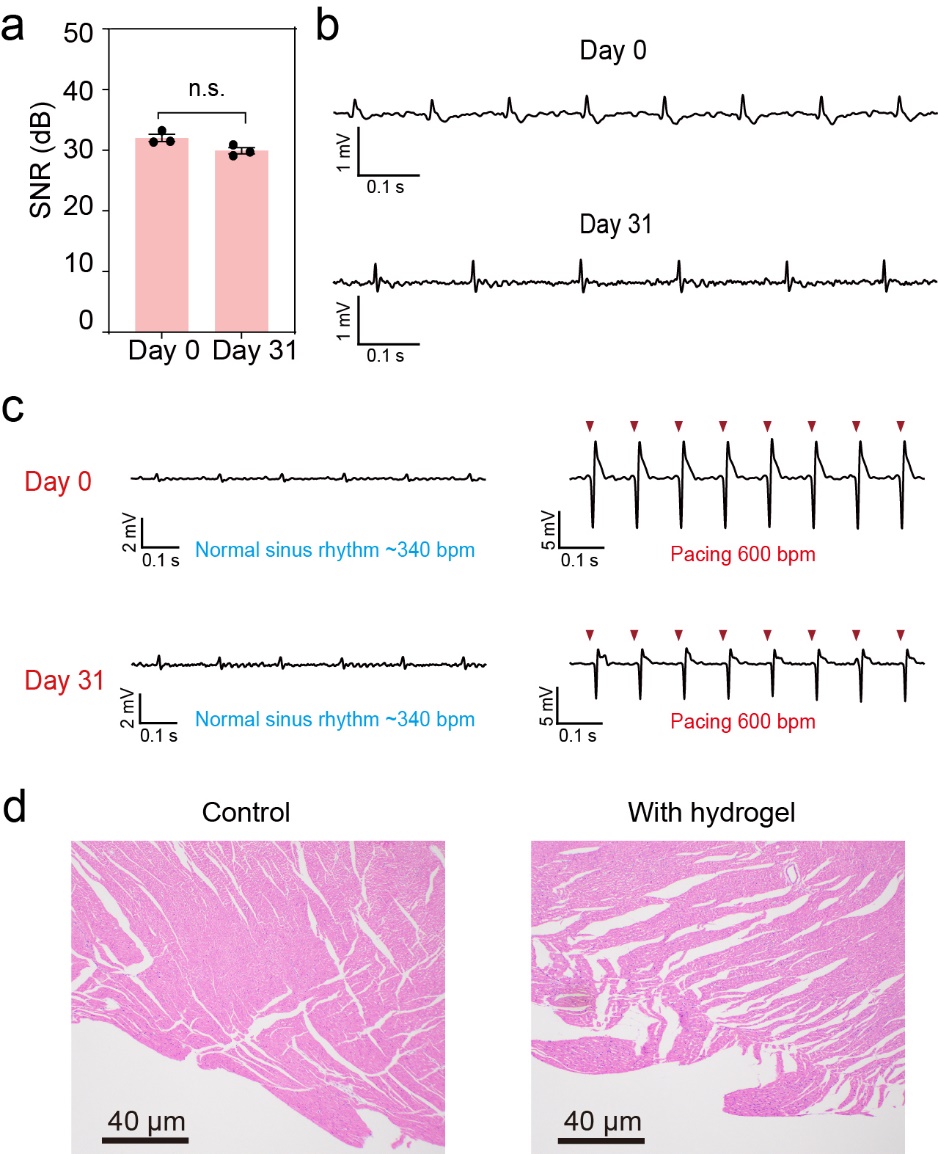


**Figure S35.** a) The comparison of the SNR values of the ECG signals recorded by the patch on day 0 and day 31 (*n*=3). b) ECG signals were detected using hydrogel electrodes on day 0 and day 31 after implantation (*n*=3). *P* value is 0.188. c) ECG signals captured by Powerlab during cardiac pacing with hydrogel electrodes on day 0 and day 31 (*n*=3). d) Representative histology images stained with hematoxylin and eosin without and with hydrogel electrodes in rat hearts after 14 days of implantation (*n*=3).


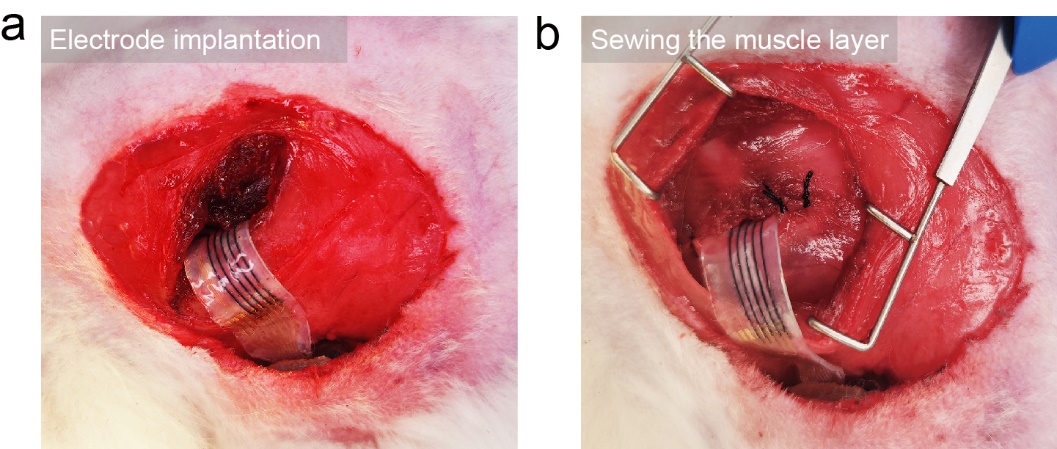


**Figure S36.** Surgical procedure for implanting the WSPD in rabbits. a) The electrodes are implanted in the subcutaneous thoracic cavity and the WSPD is buried under the skin. b) Sutures are applied to the rib cage layer (*n*=3).


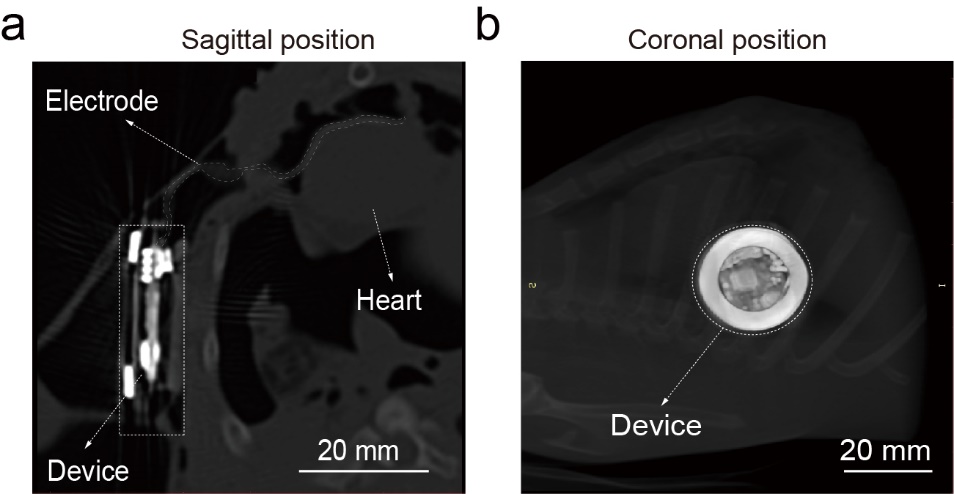


**Figure S37.** CT images of a rabbit with a WSPD implanted. a) The image represents the sagittal view of the WSPD. b) The image represents the coronal view of the WSPD.


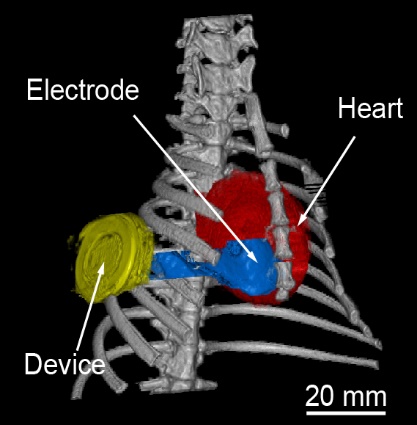


**Figure S38.** Three-dimensional reconstruction of CT images. The image depicts a wireless power supply device secured subcutaneously, with a hydrogel electrode connected to it. The hydrogel electrode is implanted into the chest cavity through rib interstices and adheres to the surface of the heart.

**Table S1.** Comparison of conductivity and stretchability for various electrode materials.

| Electrode | Conductivity | Stretchability | Pacing condition | Ref. |
| --- | --- | --- | --- | --- |
| Au | 4.56 × 107 S m^−1^ | 20% | Rabbit (Ex vivo) | [[1](#_ENREF_1)] |
| Pt | 9.43 × 106 S m^−1^ | 1.82 % (electrode)  5 % (device) | Rat (Ex vivo / in vivo) | [[2](#_ENREF_2)] |
| PEDOT:PSS | <10 S cm^−1^ | < 6% | N / R | [[3](#_ENREF_3)] |
| PEDOT:PSS / DMSO hydrogel | ~20 S cm^−1^ in PBS, ~40 S cm^−1^ in water | > 35% strain | N / R | [[4](#_ENREF_4)] |
| PEDOT:PSS / PVA hydrogel | 9 S m^−1^ | 350% | Rat (In vivo / ex vivo) | [[5](#_ENREF_5)] |
| f-PANi / PVA hydrogel | 1.05 – 1.38 S·m^−1^ | ~500% | Rat (In vivo / ex vivo) | [[6](#_ENREF_6)] |
| SWCNT / BBE | 2.06 – 17.84 S m^−1^ | 400% | Skin | [[7](#_ENREF_7)] |
| CPAMC / PCA | 0.041 ± 0.017 S m^−1^ | ~270% | Pig heart (ex vivo) | [[8](#_ENREF_8)] |
| PEDOT:PSS / SBMA hydrogel | 160 S m^−1^ | 270% | Rat (In vivo / ex vivo), rabbit (In vivo) | This work |

**Table S2.** Pathological analysis of HE staining.

| Electrode | Hydrogel | Pt |
| --- | --- | --- |
| Polymorphonuclear leukocytes | 1 | 2 |
| Lymphocytes | 2 | 1 |
| Plasma cells | 1 | 1 |
| Macrophages | 1 | 1 |
| Giant cells | 0 | 0 |
| Necrosis | 0 | 0 |
| Neovascularisation | 2 | 3 |
| Fibrosis | 2 | 3 |
| Fatty infiltration | 0 | 0 |
| Total score | 14 | 16 |

# Table S3. Comparison of wireless, battery-free stimulator.

| Powering mode | Frequency | Size | Application | Ref. |
| --- | --- | --- | --- | --- |
| Triboelectric nanogenerators | N / R | 61 mm * 39 mm | Cardiac pacing | [[9](#_ENREF_9)] |
| Acoustic energy | N / R | 50 mm * 25 mm | Cardiac pacing | [[10](#_ENREF_10)] |
| Radiofrequency | 1 MHz | Length 20 mm, diameter 2.5 mm | Cardiac pacing, ECG detection | [[11](#_ENREF_11)] |
| Radiofrequency | 220 kHz | Length 120 mm, diameter 20 mm | Gastroesophageal stimulation | [[12](#_ENREF_12)] |
| Radiofrequency | 13.56 MHz | Diameter 10 mm | Cardiac pacing | [[2](#_ENREF_2)] |
| Radiofrequency | 13.56 MHz | 15 mm * 16 mm | Cardiac pacing | [[13](#_ENREF_13)] |
| Radiofrequency | 220 kHz | Diameter 30 mm | Cardiac pacing, ECG detection, biochemical detection | This work |

References

[1]M. Han, L. Chen, K. Aras, C. Liang, X. Chen, H. Zhao, K. Li, N. R. Faye, B. Sun, J.-H. Kim, W. Bai, Q. Yang, Y. Ma, W. Lu, E. Song, J. M. Baek, Y. Lee, C. Liu, J. B. Model, G. Yang, R. Ghaffari, Y. Huang, I. R. Efimov, J. A. Rogers, *Nat. Biomed. Eng.* **2020**, *4*, 997.

[2]J. Ausra, M. Madrid, R. T. Yin, J. Hanna, S. Arnott, J. A. Brennan, R. Peralta, D. Clausen, J. A. Bakall, I. R. Efimov, P. Gutruf, *Sci. Adv.* **2022**, *8*, eabq7469.

[3]D. J. Lipomi, J. A. Lee, M. Vosgueritchian, B. C. K. Tee, J. A. Bolander, Z. Bao, *Chem. Mater.* **2012**, *24*, 373.

[4]B. Lu, H. Yuk, S. Lin, N. Jian, K. Qu, J. Xu, X. Zhao, *Nat. Commun.* **2019**, *10*, 1043.

[5]Y. Xue, X. Chen, F. Wang, J. Lin, J. Liu, *Adv. Mater.* **2023**, *35*, 2304095.

[6]C. Yu, M. Shi, S. He, M. Yao, H. Sun, Z. Yue, Y. Qiu, B. Liu, L. Liang, Z. Zhao, F. Yao, H. Zhang, J. Li, *Nat. Commun.* **2023**, *14*, 6226.

[7]P. Xu, S. Wang, A. Lin, H.-K. Min, Z. Zhou, W. Dou, Y. Sun, X. Huang, H. Tran, X. Liu, *Nat. Commun.* **2023**, *14*, 623.

[8]Y. He, Q. Li, P. Chen, Q. Duan, J. Zhan, X. Cai, L. Wang, H. Hou, X. Qiu, *Nat. Commun.* **2022**, *13*, 7666.

[9]H. Ryu, H.-m. Park, M.-K. Kim, B. Kim, H. S. Myoung, T. Y. Kim, H.-J. Yoon, S. S. Kwak, J. Kim, T. H. Hwang, E.-K. Choi, S.-W. Kim, *Nat. Commun.* **2021**, *12*, 4374.

[10]P. Jin, J. Fu, F. Wang, Y. Zhang, P. Wang, X. Liu, Y. Jiao, H. Li, Y. Chen, Y. Ma, X. Feng, *Sci. Adv.* **2021**, *7*, eabg2507.

[11]S. Wang, Q. Cui, P. Abiri, M. Roustaei, E. Zhu, Y.-R. Li, K. Wang, S. Duarte, L. Yang, R. Ebrahimi, M. Bersohn, J. Chen, T. K. Hsiai, *Sci. Adv.* **2023**, *9*, eadj0540.

[12]C. Zhang, C. Pan, K. F. Chan, J. Gao, Z. Yang, K. K. C. Leung, D. Jin, Y. Wang, N. Xia, Z. Ning, X. Wang, S. Jiang, Z. Zhang, Q. Wang, B. Hao, P. W. Y. Chiu, L. Zhang, *Sci. Adv.* **2023**, *9*, eade8622.

[13]Y. S. Choi, R. T. Yin, A. Pfenniger, J. Koo, R. Avila, K. Benjamin Lee, S. W. Chen, G. Lee, G. Li, Y. Qiao, A. Murillo-Berlioz, A. Kiss, S. Han, S. M. Lee, C. Li, Z. Xie, Y.-Y. Chen, A. Burrell, B. Geist, H. Jeong, J. Kim, H.-J. Yoon, A. Banks, S.-K. Kang, Z. J. Zhang, C. R. Haney, A. V. Sahakian, D. Johnson, T. Efimova, Y. Huang, G. D. Trachiotis, B. P. Knight, R. K. Arora, I. R. Efimov, J. A. Rogers, *Nat. Biotechnol.* **2021**, *39*, 1228.
